# ARL3 GTPases facilitate ODA16 unloading from IFT in motile cilia

**DOI:** 10.1126/sciadv.adq2950

**Published:** 2024-09-04

**Authors:** Yameng Huang, Xiaoduo Dong, Stella Y. Sun, Teck-Kwang Lim, Qingsong Lin, Cynthia Y. He

**Affiliations:** ^1^ Department of Biological Sciences, National University of Singapore, Singapore, Singapore.; ^2^ Department of Structural Biology, University of Pittsburgh, Pittsburgh, PA, USA.; ^3^ The Centre for BioImaging Sciences, National University of Singapore, Singapore, Singapore.

## Abstract

Eukaryotic cilia and flagella are essential for cell motility and sensory functions. Their biogenesis and maintenance rely on the intraflagellar transport (IFT). Several cargo adapters have been identified to aid IFT cargo transport, but how ciliary cargos are discharged from the IFT remains largely unknown. During our explorations of small GTPases ARL13 and ARL3 in *Trypanosoma brucei*, we found that ODA16, a known IFT cargo adapter present exclusively in motile cilia, is a specific effector of ARL3. In the cilia, active ARL3 GTPases bind to ODA16 and dissociate ODA16 from the IFT complex. Depletion of ARL3 GTPases stabilizes ODA16 interaction with the IFT, leading to ODA16 accumulation in cilia and defects in axonemal assembly. The interactions between human ODA16 homolog HsDAW1 and ARL GTPases are conserved, and these interactions are altered in HsDAW1 disease variants. These findings revealed a conserved function of ARL GTPases in IFT transport of motile ciliary components, and a mechanism of cargo unloading from the IFT.

## Teaser

A molecular switch helps cargo release from ciliary transport machinery.

## INTRODUCTION

Defects in cilia structure and function can result in a large spectrum of diseases known as ciliopathies (*1*). In comparison to primary cilia, motile cilia have additional axonemal structures such as the inner and outer dynein arms (IDA and ODA) and the central pair (CP) microtubules that are required for ciliary beating (*2*). Multiple protein trafficking pathways are involved in cilia biogenesis, maintenance, and function. The best characterized is the intraflagellar transport (IFT) pathway, where the multimeric IFT complex mediates bidirectional protein trafficking into and out of the ciliary compartment (*3*, *4*). The IFT complex can bind directly to some cargos such as tubulin (*5*) or indirectly to other cargos via cargo adapters (*6*). For example, the cargo adapter protein ODA16 mediates IFT transport of the ODA complexes (*7*), IDA3 mediates the transport of the inner dynein arm (IDA) complexes via IFT (*8*), and the octameric adapter BBSome mediates the import and export of selected ciliary membrane proteins (*9*, *10*). While recent studies have made substantial progress in understanding the mechanism of cargo recognition by the IFT, how ciliary cargos and their adapters are released from the IFT within the ciliary compartment is largely unknown. The current model posits that IFT cargo unloading occurs near the cilia tip, likely when the IFT is remodeled from anterograde to retrograde IFT (*11*, *12*). However, live cell imaging of the IFT cargo dynein regulatory complex subunit 4 (DRC4) shows that DRC4 can be dissociated from the IFT along the cilia before reaching the tip (*11*). The ODA complex also appears to be released from the IFT immediately upon cilia entry, as its ciliary distribution does not require IFT transport to the tip (*7*). The molecular details of cargo release from the IFT are still lacking.

ARL13B and ARL3 are cilia-associated, Arf/Arl family guanosine triphosphatases (GTPases) that can be traced back to the last eukaryotic common ancestor (*13*). Mutations in ARL13B and ARL3 lead to changes in axonemal organization and ciliary membrane protein composition (*14*–*17*). The seminal discoveries of ARL3 functions in releasing lipidated cargos from carrier proteins UNC119 or PDE6δ (*18*, *19*) and ARL13B functions as a guanine exchange factor (GEF) for ARL3 (*20*) have explained the mechanisms of ARL13B and ARL3 in the transport of selected ciliary membrane proteins involved in signaling (*21*). More recent studies in *Chlamydomonas reinhardtii* have also identified a role for ARL13B and ARL3 in BBSome-mediated IFT of ciliary membrane proteins (*22*, *23*). Notably, ARL13B mutants in *C. reinhardtii* and ARL3 mutants in *Leishmania donovani* both result in flagella motility defects (*23*, *24*). The mechanisms of ARL13B and ARL3 in motile cilia biogenesis and function, however, remained elusive.

**Fig. 1.**
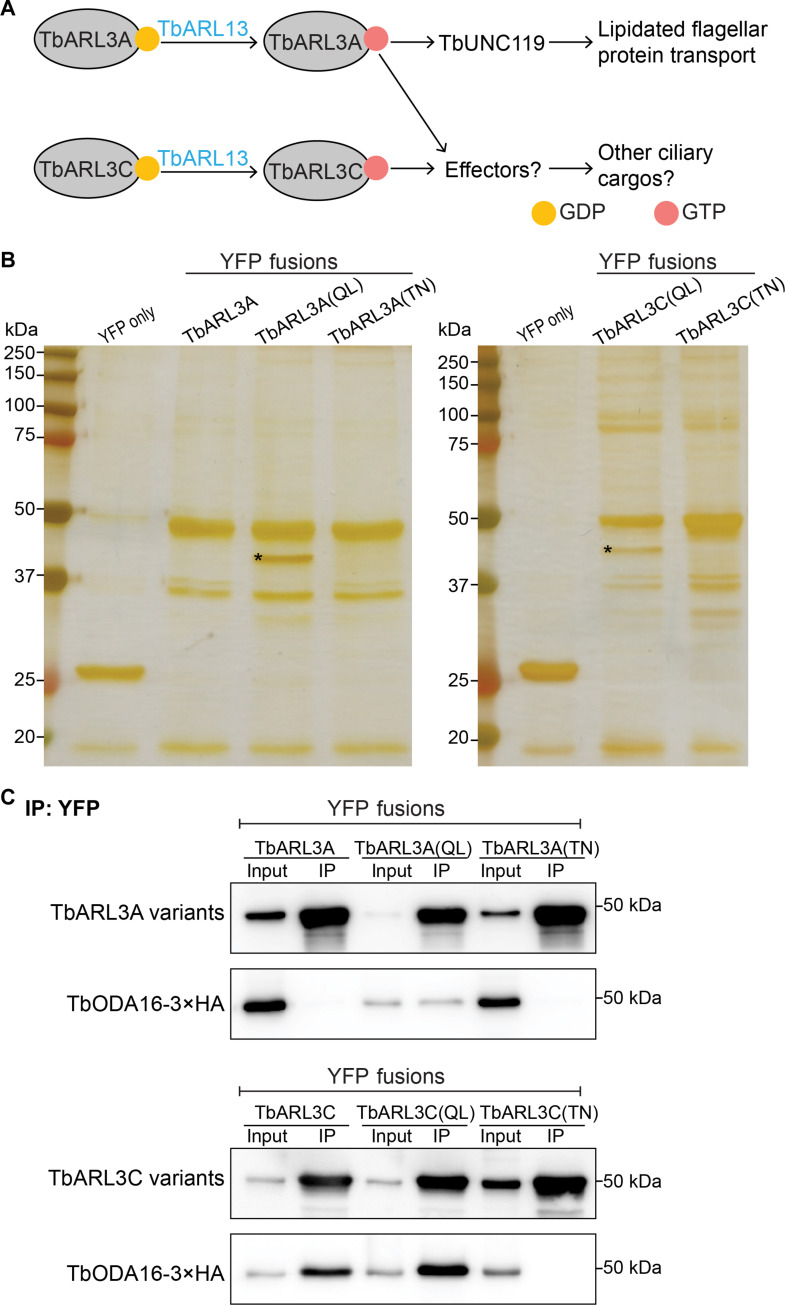
TbODA16 is an effector of both TbARL3A and TbARL3C. (**A**) Overview of the TbARL13-TbARL3 pathway in *T. brucei.* (**B**) Silver staining of proteins co-immunoprecipitated with TbARL3 variants led to the identification of TbODA16 [marked by an asterisk (*)] as an effector for TbARL3A and TbARL3C. (**C**) Co-immunoprecipitation (co-IP) experiments showing TbODA16 interaction with TbARL3A and TbARL3C in a GTP-dependent manner. YFP, yellow fluorescent protein.

Previously, we have identified a single ARL13B ortholog, TbARL13 in the flagellum of *Trypanosoma brucei* (*25*, *26*), an evolutionarily divergent protozoan parasite that causes Trypanosomiasis. Two distinct ARL3 homologs, TbARL3A and TbARL3C, interact with TbARL13. Both TbARL3A and TbARL3C can be guanosine 5′-triphosphate (GTP)–loaded by TbARL13 in guanine exchange reactions, and both have flagellar functions (*25*). The lipidated cargo carrier TbUNC119 is a specific effector of TbARL3A and functions in lipidated flagellar protein transport (Fig. 1A) (*27*). While TbARL13 is essential for flagellar biogenesis and cell survival (*25*), the depletion of TbUNC119 has no observable effects on either flagellar morphology or cell proliferation (*27*, *28*). The RNA interference (RNAi) depletion of TbARL3A or TbARL3C alone moderately affected flagellar morphology, and the cells continued to proliferate at a slower rate (fig. S1, A to C). Simultaneous RNAi of both TbARL3A and TbARL3C, however, led to cell death after 24 hours (fig. S1C), recapitulating the growth phenotype previously observed with TbARL13 RNAi (*25*). We thus hypothesized that TbARL13 has additional flagellar functions beyond TbUNC119-mediated lipidated protein transport, possibly via additional TbARL3 effectors (Fig. 1A).

## RESULTS

### TbODA16 is an effector of TbARL3A and TbARL3C

To search for potential effectors, we expressed QL and TN mutants that correspond to constitutively active and inactive forms of TbARL3A and TbARL3C as yellow fluorescent protein (YFP) fusions using a cumate-inducible system in *T. brucei* (*29*) and performed immunoprecipitation using green fluorescent protein (GFP)–Trap. One distinct band (asterisks, Fig. 1B) coprecipitated with the active TbARL3A(Q70L) and TbARL3C(Q77L), but not the inactive TbARL3A(T30N) or TbARL3C(T28N). Mass spectrometry (MS) analysis identified this band to be the protein product of Tb927.8.4210. Further bioinformatic analysis showed that Tb927.8.4210 encodes a WD repeat-containing protein that is homologous to ODA16 (outer row dynein assembly protein 16 homolog), which is also known as DAW1 (dynein assembly factor with WDR repeat domains 1) or WDR69 (WD repeat-containing protein 69). The amino acid sequence encoded by Tb927.8.4210 shares 60% identity with human DAW1 and 65% identity with *C. reinhardtii* ODA16 (fig. S2). We have therefore renamed Tb927.8.4210 to TbODA16 to reflect this homology. The GTP-dependent interactions between TbARL3 GTPases and TbODA16 were verified by co-immunoprecipitation (co-IP) analyses (Fig. 1C and fig. S1D). Wild-type (WT) TbARL3C but not TbARL3A co-immunoprecipitated efficiently with TbODA16. It is possible that TbARL3C is more abundantly present in the GTP-bound form in *T. brucei*, as TbARL3C exhibits greater intrinsic GDP-dissociation and GTP-binding activities than TbARL3A (*25*).

The interaction between TbARL3A/TbARL3C with TbODA16 is highly specific. TbARL2, which is closely related to TbARL3A and TbARL3C (*25*) and affects the microtubule-based cytoskeleton in *T. brucei* (*30*), had no detectable interaction with TbODA16 (fig. S1D). TbARL3B, another ARL3 homolog in *T. brucei*, does not interact with TbARL13 and does not exhibit any abnormal flagella phenotype when overexpressed as a constitutively active mutant (*25*). TbARL3B RNAi also did not have any detectable abnormalities on cell growth or flagella morphology (fig. S1, E to G). Hence, in this study, we focus on TbARL3A and TbARL3C for their potential regulatory functions on TbODA16.

### TbODA16 is required for axonemal assembly

ODA16 has been best characterized in *C. reinhardtii* (*7*, *31*, *32*), where it is proposed to act as an IFT cargo adapter to facilitate ciliary transport of the ODA complex (*33*). The IFT transport of ODA intermediate chain 2 (IC2) was reduced in a *C. reinhardtii* ODA16 mutant (*34*). ODA16 interacts with IFT46, and both ODA16 and IFT46 are involved in the targeting of the ODA complex to the flagella (*7*, *35*).

**Fig. 2.**
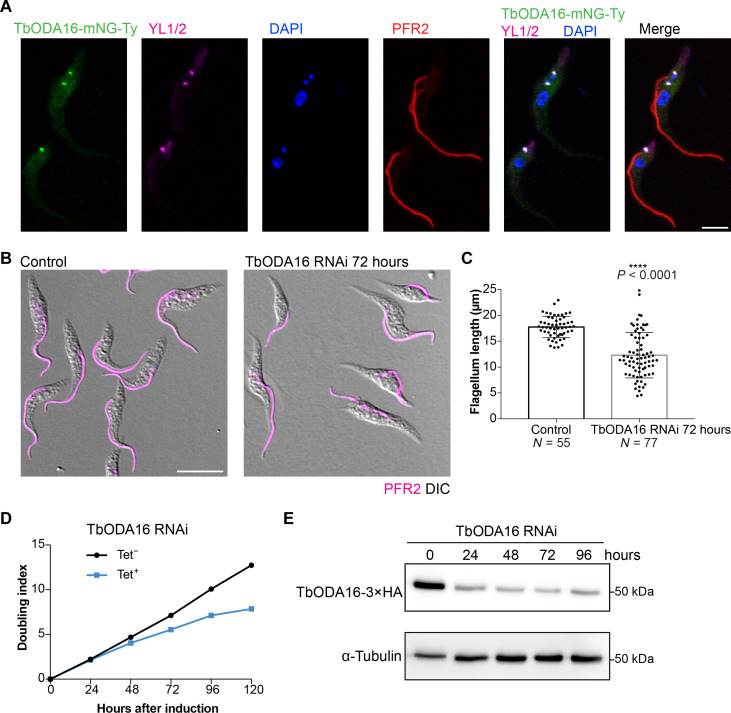
TbODA16 is essential for *T. brucei* cell proliferation and cilia biogenesis. (**A**) Immunofluorescence of cells stably expressing TbODA16 fusion with mNeonGreen(mNG) and Ty tag from an endogenous allele. The cells were costained with anti-YL1/2 for the basal bodies, anti-PFR2 for the flagella, and 4′,6-diamidino-2-phenylindole (DAPI) for the DNA containing nuclei (large ovals) and kinetoplasts (small dots). Scale bar, 5 μm. (**B**) Immunofluorescence of control and TbODA16 RNAi cells stained for the flagellum (PFR2). Scale bar, 10 μm. (**C**) Flagellum length measurement of cells shown in (B). The results were shown as means ± SD. *P* values were calculated by unpaired *t* test with Welch’s correction. ****P < 0.0001. (**D**) Growth assays of control (Tet^─^) and TbODA16 RNAi (Tet^+^) cells. (**E**) Immunoblots showing depletion of endogenously tagged TbODA16-3×HA protein upon induction of TbODA16 RNAi with tetracycline (Tet; 10 μg/ml).

**Fig. 3.**
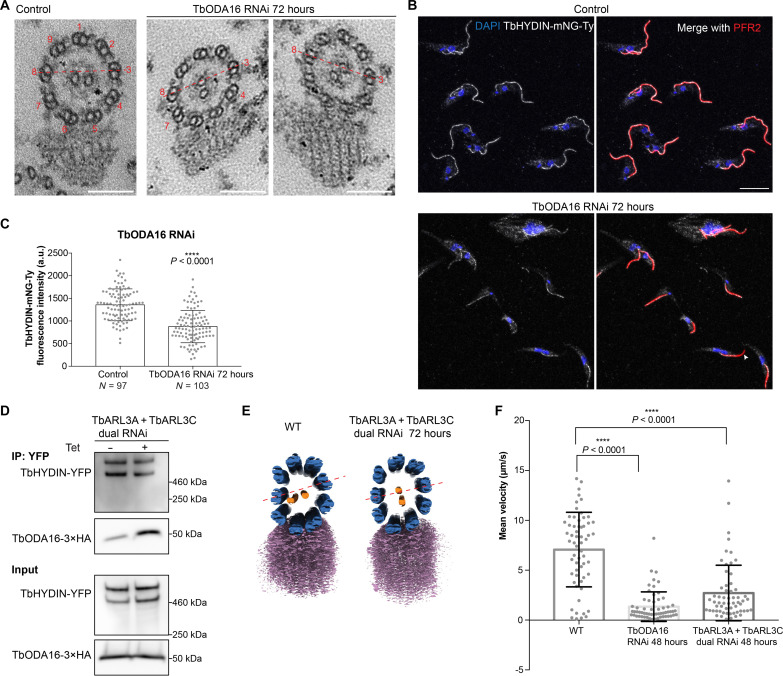
TbODA16, TbARL3A, and TbARL3C are required for proper CP microtubule orientation. (**A**) Representative TEM images showing altered CP microtubule position in TbODA16 RNAi cells. Scale bars, 100 nm. (**B**) TbODA16 RNAi was induced in cells stably expressing mNG-Ty fusion to TbHYDIN. The flagellum was stained with anti-PFR2. Scale bar, 10 μm. The arrow marked the loss of TbHYDIN in the distal region of some flagella. The flagellar intensity of TbHYDIN-mNG-Ty was measured along the distal 1.5 μm and shown in (**C**). The results were shown as means ± SD. *P* values were calculated by unpaired *t* test with Welch’s correction. a.u., arbitrary unit. (**D**) Cells stably expressing HA-tagged TbODA16 and YFP-tagged TbHYDIN were induced with tetracycline for TbARL3A/TbARL3C dual RNAi for 48 hours or not. Immunoprecipitation of TbHYDIN-YFP using GFP-Trap to assess interaction with TbODA16-3×HA. (**E**) Representative cryo–electron tomography reconstructions showing CP microtubule (orange) misalignment in TbARL3A/TbARL3C dual RNAi cells. Microtubule doublets and PFR are shown in blue and purple, respectively. (**F**) Mean velocity was measured for WT, TbODA16 RNAi, and TbARL3A/TbARL3C RNAi cells (*n* = 60 cells, each) based on time-lapse movies shown in movies S1 to S3. The results were shown as means ± SD. *P* values were obtained from one-way analysis of variance (ANOVA) with Tukey’s multiple comparisons test. ****P < 0.0001.

In *T. brucei*, TbODA16 fusion to mNeonGreen was enriched at the basal bodies (Fig. 2A) (*36*). The RNAi silencing of TbODA16 led to reduced flagellar length (Fig. 2, B and C), slowed cell proliferation in culture (Fig. 2, D and E), and paralyzed cell motility (movies S1 and S2). To further understand the effects of TbODA16 on flagellar assembly, we processed TbODA16 RNAi cells for transmission electron microscopy (TEM) (Fig. 3A). In trypanosome flagellum, the microtubule axoneme is stably associated with a paraflagellar rod (PFR) complex via microtubule doublets 4 to 7 (*37*), providing a convenient positional reference. The orientation of the CP microtubules is fixed, and they always align approximately with doublets 3 and 8 (*37*). In TbODA16 RNAi cells, however, the orientation of the CP microtubules became highly variable. Apart from the CP microtubules, the overall “9 + 2” axonemal structure appeared intact in TbODA16 RNAi cells (Fig. 3A).

To test if TbODA16 depletion may have changed the molecular composition of the ODA complex, the ODA intermediate chains TbIC1 (also known as TbDNAI1) and TbIC2 (*38*) were tagged with mNeonGreen and expressed endogenously from their native alleles. While TbIC2 localized to the flagellum in both control and TbODA16 RNAi cells without discernible difference (fig. S3, A and B), TbIC1 intensity along the flagellum was partially reduced in RNAi cells (fig. S3, C and D). It is unclear why TbODA16 RNAi affected TbIC1 but not TbIC2. The partial reduction of flagellar TbIC1 and unchanged TbIC2 in TbODA16 RNAi cells, however, are consistent with the TEM observation that the ODA complex was generally intact in these cells (Fig. 3A). Similarly in *C. reinhardtii*, zebrafish and human, ODA16 facilitates efficient ODA trafficking, but ODA16 deficiency does not completely inhibit ODA import into the cilia (*7*, *39*, *40*). ODA16-independent transport of ODA complex may be present.

The main defect observed in TbODA16 RNAi cells was CP misalignment. TbHYDIN, a known CP component with a role in CP alignment (*41*), was reduced in the flagella upon TbODA16 RNAi (Fig. 3, B and C). TbHYDIN-YFP co-immunoprecipitated with TbODA16-3×HA (Fig. 3D), supporting the notion that TbHYDIN is a cargo of TbODA16. The effects of ODA16 on CP alignment have not been reported in other organisms, likely because the CP position is harder to analyze in cilia without a para-axonemal marker such as the PFR, and the CP is observed to rotate relative to the nine microtubule doublets in some cells including *C. reinhardtii* (*42*) and *Paramecium* (*43*). Together, our results supported a role of TbODA16 in motile cilia assembly, affecting cell motility and the axonemal localization of TbIC1 and TbHYDIN. The cellular function of ODA16 in motile cilia biogenesis, as previously reported in green algae, zebra fish, and mammalian cells, is thus also conserved in *T. brucei*.

### TbARL3A and TbARL3C regulate ODA16-IFT interaction

**Fig. 4.**
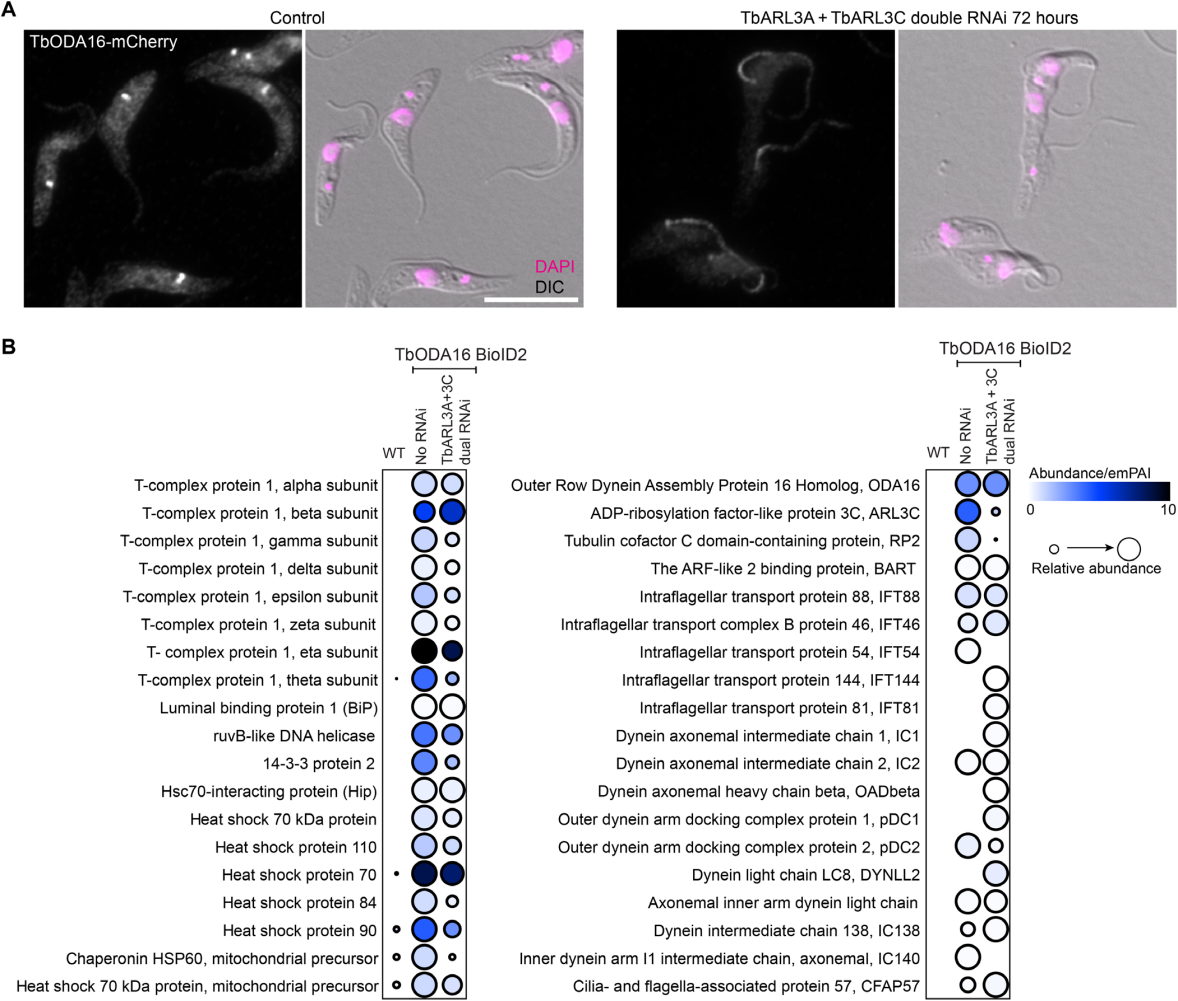
TbARL3 GTPases affect TbODA16 localization and proximity interaction. (**A**) Immunofluorescence of cells stably expressing TbODA16-mCherry before and after induction for TbARL3A/TbARL3C dual RNAi. Scale bar, 10 μm. DIC, differential interference contrast. (**B**) Comparison of BioID2-based proximity interactomes of TbODA16 before and after TbARL3A/TbARL3C dual RNAi.

Both TbARL3A and TbARL3C interacted with TbODA16 in a GTP-dependent manner (Fig. 1C and fig. S1D). Dual RNAi of TbARL3A and TbARL3C also led to CP misalignment (Fig. 3E) and impaired cell motility (Fig. 3F and movie S3), supporting functional connections between TbARL3 GTPases and TbODA16. In control cells, TbODA16 was present at the basal bodies, with some weak signals in the cytoplasm and the ciliary lumen (Fig. 4A). Upon TbARL3A/TbARL3C dual RNAi, TbODA16 became enriched in the cilia. The ciliary accumulation of TbODA16 was also observed in cells depleted of TbARL3C alone, but not in cells lacking TbARL3A (fig. S4, A to C). As an IFT cargo adapter, ODA16 is expected to eventually dissociate from the IFT and recycle to the ciliary base for another round of cargo transport. Thus, the ciliary accumulation of ODA16 suggested a possible role of TbARL3A and TbARL3C in regulating ODA16 interaction with the IFT. To investigate this possibility, we performed proximity-based BioID in cells with or without TbARL3A and TbARL3C using TbODA16-BioID2 as a bait.

**Fig. 5.**
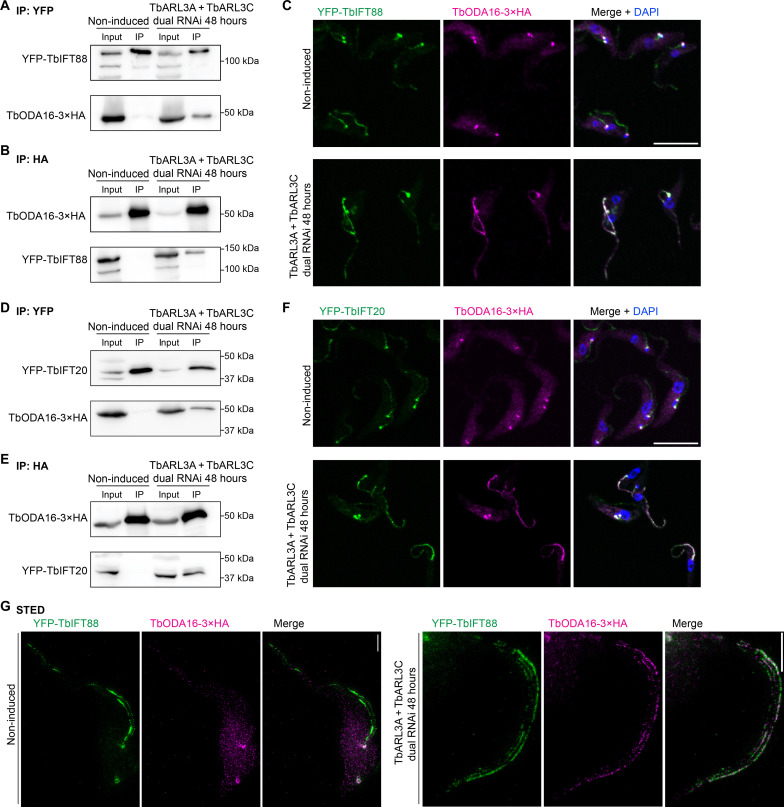
The absence of TbARL3A and TbARL3C stabilizes TbODA16 interaction with IFT. (**A** to **F**) Cells stably expressing HA-tagged TbODA16 and YFP-tagged IFT subunits IFT88 (A to C) and IFT20 (D to F), respectively, were either uninduced or induced for TbARL3A/TbARL3C dual RNAi. TbODA16-IFT interaction was assessed by co-IP using beads targeting YFP (A and D) or HA (B and E) tags. Immunofluorescence showing TbODA16 and IFT components in control and TbARL3A/TbARL3C dual RNAi cells (C and F). Scale bars, 10 μm. (**G**) STED super-resolution images of TbODA16-3×HA and YFP-TbIFT88 in control and TbARL3A/TbARL3C dual RNAi cells. Scale bars, 2 μm.

Several IFT subunits including TbIFT46, IFT81, and IFT144 were found more abundantly associated with TbODA16 in TbARL3A/TbARL3C dual RNAi cells (Fig. 4B), suggesting enhanced TbODA16-IFT interaction in the absence of the TbARL3 GTPases. To test this, TbIFT88, TbIFT46, TbIFT20, and TbIFT81 were each tagged endogenously with YFP in cells coexpressing TbODA16-3×HA at the native level. These cells were then induced for TbARL3A/TbARL3C dual RNAi or not, and co-IPs were performed using anti–hemagglutinin (HA) or GFP-Trap beads (Fig. 5 and fig. S5). In all cases, TbODA16-IFT interaction was stronger in cells depleted of TbARL3A and TbARL3C. Consistently, TbODA16 was mainly present at the basal bodies in control cells; upon depletion of TbARL3 GTPases, TbODA16 became enriched in the cilia, colocalizing with the IFT components (Fig. 5 and fig. S5). Partial colocalization of TbODA16 with IFT88 in TbARL3A/TbARL3C dual RNAi cells was also confirmed by stimulated emission depletion (STED) super-resolution imaging (Fig. 5G).

As both TbARL3A and TbARL3C can be regulated by TbARL13 (*25*) (Fig. 1A), the effects of TbARL13 on TbODA16 were also examined. TbARL13 RNAi led to TbODA16 accumulation in the flagellum, colocalizing with TbIFT88 (fig. S6A). TbODA16 expression levels were not affected (fig. S6B). On the basis of these results, we concluded that TbARL13, TbARL3A, and TbARL3C all regulate TbODA16 interaction with the IFT train.

### Active TbARL3A and TbARL3C can displace TbODA16 from the IFT

**Fig. 6.**
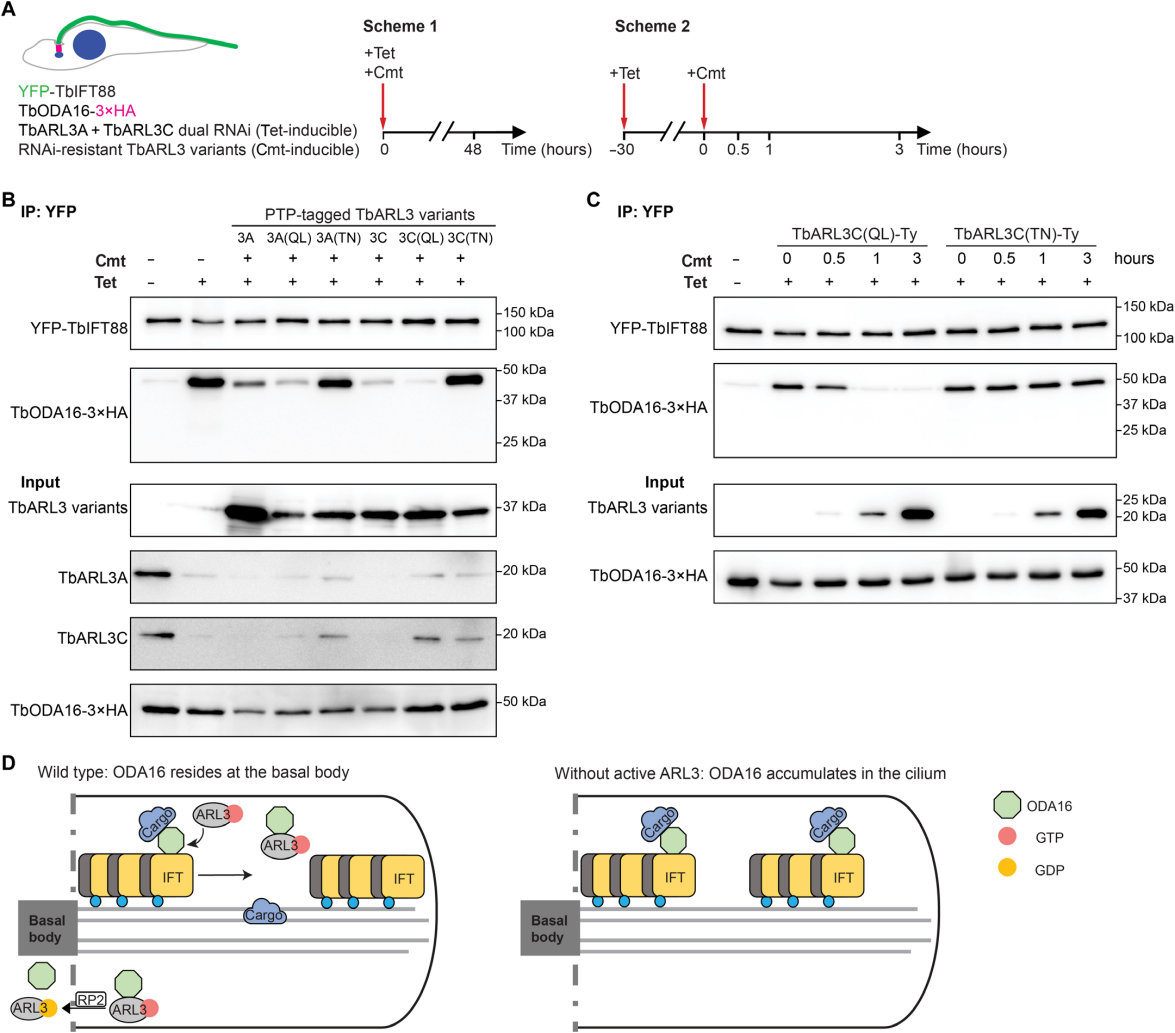
Active TbARL3A and TbARL3C displace TbODA16 from IFT. (**A**) Schematics of the displacement assays. (**B**) Co-IP demonstrating the effects of RNAi-resistant TbARL3 variants on TbODA16-IFT interactions following scheme 1. Tet concentration: 10 μg/ml. Cumate (Cmt) concentration: 10 μg/ml. (**C**) Co-IP experiments following scheme 2 demonstrating rapid displacement of TbODA16 from the IFT by active TbARL3C. Cmt concentration: 20 μg/ml. (**D**) Model of ARL3 in ODA16-mediated IFT based on the current study. In WT cells, ODA16 facilitates ciliary cargo transport by IFT. Upon entry into the cilia, active ARL3 binds to ODA16 and displaces ODA16 from the IFT. Hydrolysis of ARL3•GTP by RP2 (*90*), a known ARL3 GTPase-activating protein, helps to recycle ODA16 for another round of cargo transport. In cells without active ARL3, ODA16 remains bound to the IFT.

To test if active TbARL3A and TbARL3C could displace TbODA16 from the IFT, the TbARL3A/TbARL3C dual RNAi cells with stable expression of TbODA16-3×HA and YFP-tagged TbIFT88 were further engineered to include cumate-inducible expression of RNAi-resistant TbARL3 variants. Co-IP was then performed using GFP-Trap, and the amount of TbODA16 associated with IFT was evaluated before and after TbARL3 variants were induced (Fig. 6). In scheme 1, expressions of TbARL3A and TbARL3C variants were induced at the same time of RNAi induction. TbODA16-IFT interaction was barely detectable in cells expressing TbARL3A and TbARL3C, either WT or QL forms, demonstrating functional rescue by the RNAi-resistant, active TbARL3 variants. The expression of the inactive TN mutants, however, did not reduce TbODA16-IFT interaction (Fig. 6B). In scheme 2, TbARL3A/TbARL3C dual RNAi was first induced for 30 hours, which stabilized TbODA16-IFT interaction (Fig. 6C). The expression of TbARL3C variants was then induced in the cell system. TbARL3C(QL) rapidly displaced TbODA16 from the IFT within 1 hour of induction. In contrast, TbARL3C(TN) that was expressed at similar levels to TbARL3C(QL) did not affect TbODA16-IFT interaction.

### HsDAW1, the human homolog to ODA16, interacts with IFT88 and small GTPases ARL2 and ARL3

**Fig. 7.**
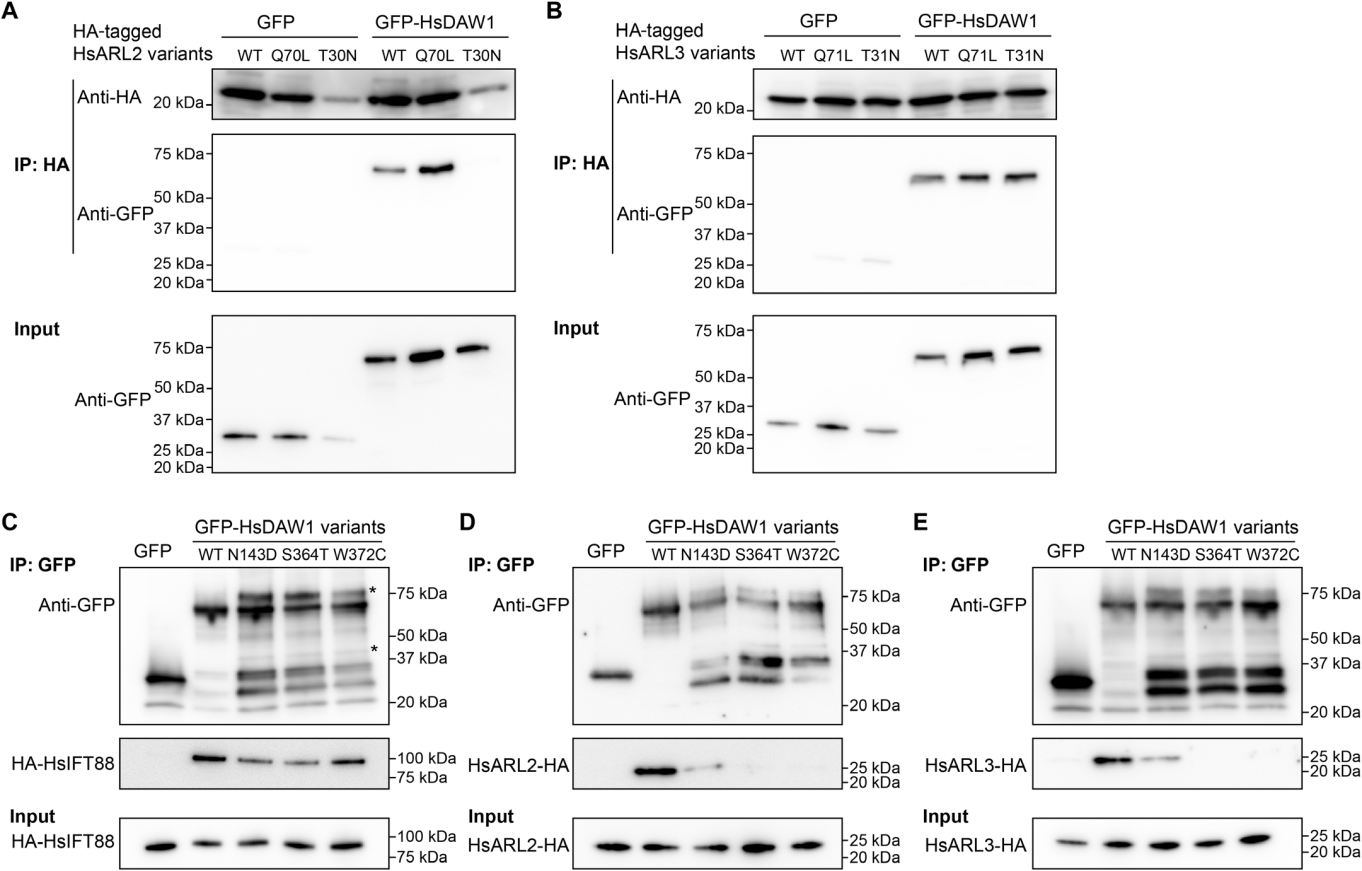
DAW1 interactions with ARL2, ARL3 and IFT components are conserved in human cells. (**A**) HA-tagged HsARL2, HsARL2(Q70L), or HsARL2(T30N) variants were coexpressed with GFP or GFP-HsDAW1 in HEK293T cells. Immunoprecipitation of HsARL2-HA variants using anti-HA IP resin were used to assess interaction with GFP-HsDAW1. Poor solubility of HsARL2(T30N)-HA led to less binding to the resin. (**B**) HA-tagged HsARL3, HsARL3(Q71L), or HsARL3(T31N) variants were coexpressed with GFP or GFP-HsDAW1 in HEK293T cells. Immunoprecipitation of HsARL3-HA variants using anti-HA IP resin were used to assess interaction with GFP-HsDAW1. (**C** to **E**) GFP or GFP-HsDAW1 disease variants were coexpressed with HA-HsIFT88 (C), HsARL2-HA (D), and HsARL3-HA (E) in HEK293T cells. Immunoprecipitation by GFP-Trap was used to assess the interaction. GFP was used as a negative control in all immunoprecipitation analyses. Extra protein bands [marked by an asterisk (*)] may result from protein degradation and ubiquitination.

In vertebrates, ARL13 and ARL3 GTPases have been mostly studied for their signaling functions in cells containing primary cilia. ODA16, however, is exclusively found in organisms with motile cilia (*44*). Therefore, the interaction between ODA16 and ARL3 GTPase had not been recognized nor investigated in any cilia models. To test if the interaction between ODA16 and ARL GTPase is conserved in mammalian cells, HsDAW1 was expressed in human embryonic kidney (HEK) 293T cells together with HsARL3 or HsARL2, which is closely related to HsARL3 (*45*). HsDAW1 interacted with HsARL2 in a GTP-dependent manner (Fig. 7A), but HsDAW1 interaction with HsARL3 was not affected by GTP (Fig. 7B). Furthermore, HsDAW1 interacted with HsIFT88 when both proteins were ectopically expressed in HEK293T cells (Fig. 7C), consistent with HsDAW1 function as an IFT cargo adaptor. Direct interaction between ODA16 and IFT46 is observed in *C. reinhardtii* (*31*), but this interaction is not conserved in humans (*46*). Our results suggest that HsDAW1 may interact with the IFT complex via HsIFT88, but direct interaction between HsDAW1 and HsIFT88 is yet to be experimentally verified.

Several HsDAW1 missense mutations have been found in patients with motile ciliopathy or congenital heart disease (CHD), where ODA assembly or ciliary beating is compromised (*40*, *47*). Three HsDAW1 disease mutants—N143D, S364T, and W372C—have also been studied in zebrafish. None of these mutants is able to rescue cilia motility defects observed in *daw1* mutant (*40*). We expressed N143D, S364T, and W372C mutants as GFP fusions in HEK293T cells. The interaction between HsARL2/HsARL3 and HsDAW1 was abolished in S364T and W372C mutants and much reduced in N143D (Fig. 7, D and E). However, the interaction between HsIFT88 and HsDAW1 mutants appeared similar to the WT HsDAW1 (Fig. 7C). These results further attested the conservation and the functional importance of the interactions between ODA16 and ARL GTPases in human cells.

## DISCUSSION

In this study using *T. brucei* as a motile cilia model, we identified ODA16 as a primary effector of ARL3 GTPases. We propose that active ARL3 variants interact with ODA16 and dissociate ODA16 from the IFT, resulting in ciliary cargo unloading from the IFT. In the absence of active ARL3, cargo adaptor ODA16 cannot be dissociated from the IFT, resulting in its accumulation in cilia (Fig. 6D). ODA16 likely dissociates from the IFT near the proximal region of the cilia, where *T. brucei* ARL13 is enriched (*25*), allowing released ODA16-ARL3 to be immediately recycled to the ciliary base and used for another round of cargo transport (Fig. 6D). ODA16 cargos are likely released from ODA16 at the same time, as TbHYDIN-TbODA16 interaction was strengthened in cells depleted of TbARL3 GTPases (Fig. 3D). It is not known if additional factors may be required to dissociate the axonemal cargos from ODA16.

Both TbARL3A and TbARL3C, in their respective active forms, were able to regulate ODA16-IFT interactions. However, it appeared that TbARL3C plays a major regulatory role in this regard given its stronger binding to TbODA16 (Fig. 1C) and more pronounced effect on TbODA16 ciliary accumulation upon RNAi (fig. S4, A and B). TbARL3A, in addition to its role in TbUNC119-mediated lipidated protein transport, may partially complement TbARL3C in regulating ODA16-IFT transport. Our results thus demonstrated that in *T. brucei*, ARL13 functions through two distinct ARL3 forms, regulating both lipidated flagellar protein transport and IFT transport of motile ciliary components.

Despite extensive efforts using different tags and different expression systems, purified TbODA16 and individual IFT subunits were highly unstable, making it difficult to test the displacement model in vitro. ODA16 is recently classified as one of the dynein axonemal assembly factors (DNAAFs), which assist the cytoplasmic pre-assembly, maturation, and ciliary transport of the axonemal dynein complexes (*48*). In TbODA16-BioID, we found TbODA16 to be associated with several motile ciliary components including TbIC1 and TbIC2 (Fig. 4B). However, ODA16 RNAi has more pronounced effects on CP alignment than ODA assembly. Despite near intact ODA structure, ODA16 RNAi cells exhibited strong motility defects. It is thus possible that ODA16 has a broader effect on ciliary motility, not limited to its effect on ODA. Further examination of TbODA16-BioID candidates will help to identify axonemal cargos of TbODA16.

TbODA16 was also associated with many chaperone proteins, including all eight subunits of the T-complex protein ring complex (TRiC) (Fig. 4B and fig. S7A). The interaction between TbODA16 and TRiC was confirmed by co-IP (fig. S7B). TRiC is responsible for the folding of ~10% of the proteome including tubulin and actin and assists in complex formation in various eukaryotic organisms. It thus appears that additional cellular factors, particularly chaperones, are required for the formation or stabilization of TbODA16 in complex with IFT and/or the axonemal cargos.

Mechanistic studies of ARL13B and ARL3 have been mostly performed on primary cilia, focusing on their sensory and developmental functions. In lipidated intraflagellar transport (LIFT) (*21*), active ARL3 displaces lipidated cargos allosterically from LIFT carriers UNC119 or PDE6δ (*18*, *19*). However, it is not clear whether LIFT requires IFT. In our study, we showed that active ARL3 binds to ODA16 and displaces this cargo adapter from the IFT. This point of action is distinct to the previously established mechanism of ARL3 in LIFT. Although the structural detail of ARL3 in IFT cargo release is still lacking, our observations suggest functional flexibility and diversity of ARL3 GTPases as displacement factors in both LIFT and IFT.

Besides *T. brucei*, we also provided evidence supporting the conservation and functional importance of ODA16 interaction with small GTPases ARL2/ARL3 and IFT in human cells. Notably, three HsDAW1 disease mutants showed reduced interaction with ARL2/ARL3, which may explain the motile cilia and laterality defects observed in patients. It has long been noted that besides high sequence similarity, HsARL2 and HsARL3 have common effectors such as UNC119 (*49*, *50*) and common regulators such as the GTPase activating proteins ELMODs (*51*) and function similarly in lipidated cargo transport (*19*, *45*). It is possible that the IFT cargo unloading function we observed for *T. brucei* ARL3 GTPases is also conserved in human but is additionally regulated by the closely related HsARL2. Our results can also explain the observed cilia effects of vertebrate ARL2 and ARL2-binding protein ARL2BP [aka BART, an ARL2 effector and a co-GEF for ARL3 (*52*)]. Depletion of either protein leads to structural defects of photoreceptor cilia (*53*, *54*). Patients with ARL2BP mutations and animals with ARL2BP knockout also exhibit defects in motile cilia, affecting spermatogenesis and left-right patterning (*55*). Although HsARL3 interaction with HsDAW1 appeared independent of GTP, our current results cannot exclude a role of HsARL3 in ODA assembly. Notably, a human homolog to Shulin, a *Tetrahymena* DNAAF required for ODA packaging (*56*), was found to interact with ARL3 in mammalian cells containing primary cilia (*50*). The significance of this interaction and whether this interaction also occurs in motile cilia are yet to be determined.

Besides ARL13B and ARL3 that are involved in human Joubert syndrome, the IFT complex, HYDIN, and the ODA complex are all confirmed human ciliopathy genes (*1*). Recent analyses of ODA16 in mice and humans also demonstrate a link of this gene to primary ciliary dyskinesia and CHDs (*40*, *57*, *58*). Our findings established a functional link between ARL13B and the IFT pathway via an ARL3 effector ODA16, explaining the essential and diverse roles of ARL13B in ciliary transport of both membrane proteins required for signaling and axonemal cargos important for motility and providing insights to the disease mechanisms of ARL13B-ARL3 in motile ciliopathies.

## MATERIALS AND METHODS

### Cell culture

Procyclic form of *T. brucei* that proliferates in tsetse fly midgut was used throughout this study. The cells were cultured in Cunningham’s medium (*59*) supplemented with 10% heat-inactivated fetal bovine serum (Hyclone or Gibco) at 28°C. The cell line DIY (double-inducible YTat1.1) was engineered by stable transfection of YTat1.1 cells (*60*) with a pSmOxNUS vector that enables tetracycline- and cumate-inducible expression (*29*). *T. brucei* strain 427 29-13 (*61*), a tetracycline-inducible cell line, was used in TbARL13 RNAi experiments. To select and maintain stable transfectants, the following antibiotic concentrations were applied: geneticin [15 μg/ml (for maintenance) or 60 μg/ml (for selection)], hygromycin (50 μg/ml), puromycin (5 μg/ml), blasticidin (10 μg/ml), phleomycin (5 μg/ml). Unless otherwise stated, tetracycline (10 μg/ml) and cumate (10 μg/ml) were used to induce gene expressions. HEK293T cells were obtained from American Type Culture Collection and cultured in Dulbecco’s modified Eagle’s medium supplemented with 10% fetal bovine serum at 37°C with 5% CO_2_.

### Transfection

For stable transfection of *T. brucei*, ~5 × 10^7^ cells were washed once with 5 ml of cytomix [2 mM EGTA (pH 8.0), 120 mM KCl, 0.15 mM CaCl_2_, 10 mM K_2_HPO_4_, 5 mM MgCl_2_, and 25 mM Hepes (pH 7.6), modified from (*62*)]. The cells were then resuspended with 0.5 ml of cytomix and combined with 15 μg of linearized DNA. The mixture was transferred to a 0.4-cm electroporation cuvette (Bio-Rad) and electroporated twice at 1500 V, 25 μF, ∞ ohms with 10-s interval using a Bio-Rad Gene Pulser. Clones of stable transfectants were obtained by serial dilution and antibiotic selection for 12 to 14 days. For transient transfection of HEK293T cells, plasmids were incubated with polyethylenimine mixture at room temperature for 15 min, transferred to HEK293T cells with 60% confluency, and incubated at 37°C with 5% CO_2_ for 48 hours.

### Plasmids

For cumate-inducible expression, the coding sequence of protein-of-interest fused to specified reporter was cloned in pDEX-CuO vectors (*29*). BioID2 was amplified from MCS-BioID2-HA (Addgene, plasmid #74224) (*63*). The PTP tag (ProtC-TEV-ProtA) consists of protein C fusion to protein A, separated by a TEV cleavage site. All constructs listed below are named after the gene, the reporter that each construct contains, and the selectable antibiotics used to generate the following stable transfectants: TbARL3A-YFP (geneticin), TbARL3A(Q70L)-YFP (geneticin), TbARL3A(T30N)-YFP (geneticin), TbARL3C-YFP (geneticin), TbARL3C(Q77L)-YFP (geneticin), TbARL3C(T28N)-YFP (geneticin), TbODA16-5×GS-3×HA-BioID2 (geneticin), TbODA16-5×GS-YFP (geneticin), GFP-Ty (blasticidin), TbARL3AiR-PTP (hygromycin), TbARL3AiR(Q70L)-PTP (hygromycin), TbARL3AiR(T30N)-PTP (hygromycin), TbARL3CiR-PTP (hygromycin), TbARL3CiR(Q77L)-PTP (hygromycin), TbARL3CiR(T28N)-PTP (hygromycin), TbARL3CiR(Q77L)-Ty (hygromycin), TbARL3CiR(T28N)-Ty (hygromycin), PTP-TbARL2 (blasticidin), PTP-TbARL2(Q70L) (blasticidin), PTP-TbARL2(T30N) (blasticidin), TbARL3A-PTP (geneticin), TbARL3A(Q70L)-PTP (geneticin), TbARL3A(T30N)-PTP (geneticin), TbARL3C-PTP (geneticin), TbARL3C(Q77L)-PTP (geneticin), and TbARL3C(T28N)-PTP (geneticin). For cumate-inducible expression of RNAi-resistant (iR) TbARL3 variants, RNAi-resistant TbARL3A and TbARL3C sequences were designed by Synonymous Mutation Generator (*64*). Synthetic *TbTCP-1-zeta* (amino acids 376 to 544), *TbARL3A*iR, and *TbARL3C*iR sequences are shown in table S1.

For tetracycline-inducible RNAi, RNAi-targeted sequences (shown in the brackets) were chosen from the RNAit server (https://dag.compbio.dundee.ac.uk/RNAit/) (*65*). The target sequence was then cloned into the p2T7 vector with phleomycin resistance (*66*). The RNAi constructs used in this study are: *TbARL3A* (nt 56 to 465), *TbARL3C* (nt 38 to 458), *TbARL3C* (nt 38 to 458)-*TbARL3A* (nt 56 to 465) for TbARL3A/TbARL3C dual RNAi, *TbARL3B* (nt 139 to 547), *TbODA16* (nt 536 to 1126), and *TbARL13* (nt 160 to 593) (*25*).

For endogenous tagging, desired amplicons were polymerase chain reaction (PCR)–amplified using the pPOT vectors as templates (*67*). Using pPOTv6 and v7 vectors (kind gifts from K. Gull, S. Dean, and J. Sunter) as templates, the following templates were constructed: pPOTv7-g418-mCherry-g418, pPOTv7-g418-YFP-g418, pPOTv7-blast-3×HA-blast, pPOTv7-hygro-3×HA-hygro, pPOTv7-puro-3×HA-puro, pPOTv6-blast-3Ty::mNG::3Ty-blast, pPOTv6-hygro-3Ty::mNG::3Ty-hygro. TbTCP-1-zeta (amino acids 1 to 375)::Strep-Tag II::CBP::HA::TbTCP-1-zeta (synthetic amino acids 376 to 544)–hygro–3′ untranslated region (nt 1 to 861) was constructed by overlapping PCR for endogenously tagging of TbTCP-1-zeta-HA.

Tags and drug-resistant genes from pPOT series were integrated to the pMOTag vector backbone (*68*) to generate pMOCtag and pMONtag vectors, which can be used for plasmid-based in situ tagging with longer homologous regions. The amplicons for pMOCTag-TbARL3A-mNG-Ty (blasticidin) and pMOCTag-TbARL3B-mNG-Ty (blasticidin) contain ~500 base pairs of homologous regions. For constitutive overexpression, pXS2-YFP (blasticidin) (*69*) was used.

The template for HsDAW1 was obtained from PlasmID Repository at Harvard Medical School. The template for HsIFT88 (*70*) was a kind gift from J.-C. Liao’s group. Vectors pXJ40-GFP, pXJ40-GFP-HsDAW1, pXJ40-GFP-HsDAW1(N143D), pXJ40-GFP-HsDAW1(S364T), pXJ40-GFP-HsDAW1(W372C), pXJ40-HsARL2-3×HA, pXJ40-HsARL2(Q70L)-3×HA, pXJ40-HsARL2(T30N)-3×HA, pXJ40-HsARL3-3×HA, pXJ40-HsARL3(Q71L)-3×HA, pXJ40-HsARL3(T31N)-3×HA, and pXJ40-3×HA-HsIFT88, were generated for the expression of specified recombinant proteins in HEK293T cells.

### Cell growth assays

Growth assays were initiated with a freshly diluted culture containing 1 × 10^6^ cells/ml *T. brucei* cells. Cell density was monitored using a hemocytometer every 24 hours. The cell culture was then diluted with fresh medium to 1 × 10^6^ cells/ml to maintain the cells in exponential growth phase. Doubling index was calculated as log_2_ (*N*_t_ × *D*_f_/*N*_0_), where *N*_t_ is the cell density at a given time point, *D*_f_ is the accumulative dilution factor, and *N*_0_ is the cell density at *t* = 0.

### Primary antibodies for immunoblots

The following antibodies were commercially available: anti-HA (1:500; mouse, Santa Cruz Biotechnology, catalog no. sc-7392, RRID: AB_627809), anti-HA (1:500; mouse, Santa Cruz Biotechnology, catalog no. sc-7392 horseradish peroxidase (HRP), RRID:AB_2894930), anti-HA (1:500; rabbit, Santa Cruz Biotechnology, catalog no. sc-805, RRID:AB_631618), anti-HA (1:2000; mouse, BioLegend, catalog no. 901501, RRID:AB_2565006), anti–protein C (1:1000; mouse, GenScript catalog no. A01774, RRID:AB_2744686), anti–α-tubulin (1:5000; mouse, Santa Cruz Biotechnology, catalog no. sc-23948, RRID:AB_628410), anti-mCherry (1:3000; rabbit, Thermo Fisher Scientific, catalog no. PA5-34974, RRID:AB_2552323), and streptavidin-HRP (1:10,000; peroxidase-conjugated streptavidin biotin-binding protein, Thermo Fisher Scientific, 21130).

Polyclonal anti-TbARL13 (1:1000; rabbit) was described previously (*25*). Monoclonal anti-Ty (1:500; mouse) was a kind gift from P. Bastin (*71*). His-PFR2, His-YFP, GST-TbARL3A(Q70L), GST-TbARL3C(Q77L), and MBP-TbODA16 proteins were expressed and purified from *Escherichia coli* (BL21). Purified proteins were used for customized antibody production by Abnova. Affinity purified antibodies were validated by immunoblots. Listed below are the immunoblot details of the customized antibodies: anti-YFP (1:1000; rabbit), anti-TbARL3A (1:250; rabbit), anti-TbARL3C (1:250; rabbit), anti-TbODA16 (1:250; rat).

### Primary antibodies for immunofluorescence assays

Anti-PFR2 (1:4000; rabbit, customized/Abnova), Anti-RFP (1:500; rabbit, Rockland catalog no. 600-401-379, RRID:AB_2209751), YL1/2 (1:2000; anti-tyrosinated α-tubulin, rat, Santa Cruz Biotechnology, catalog no. sc-53029, RRID:AB_793541), anti-HA (1:250; mouse, Santa Cruz Biotechnology, catalog no.sc-7392, RRID: AB_627809), and anti-GFP (1:1000; rabbit, Abcam catalog no. ab6556, RRID:AB_305564; used for STED only).

### Light microscopy

*T. brucei* cells were washed twice with phosphate-buffered saline (PBS, pH 7.4) before adhering to coverslips by centrifugation (2000*g*, 1 min). Cells were fixed in 4% paraformaldehyde for 10 min, permeabilized with 0.25% Triton X-100 in PBS for 5 min at room temperature, and blocked with 3% bovine serum albumin (BSA) in PBS for 30 min. The cells were then incubated with primary antibodies in the blocking buffer for 1 hour. After three washes with PBS, cells were incubated with Alexa Fluor–conjugated secondary antibodies (Invitrogen) and 4′,6-diamidino-2-phenylindole for 1 hour. Cells were washed twice with PBS and once with water before mounting to glass slides. To label the PFR with anti-PFR2 only, cells were fixed and permeabilized with cold methanol at −20°C for 5 min. Images were acquired either by a Zeiss Axio Observer Z1 fluorescence microscope with a 63×/1.4 objective or a FLUOVIEW FV3000 confocal microscope equipped with a U Plan Super Apochromat 60×/1.35 objective. STED super-resolution imaging was performed on Abberior STEDYCON built on a Nikon Ti2-E inverted microscope equipped with100×/1.45 numerical aperture oil objective lens. STAR RED and STAR ORANGE secondary antibodies (Abberior) were used for STED microscopy.

### Time-lapse imaging

To track cell movement, 15 μl of live cells in culture medium was added to a disposable hemocytometer and imaged using Zeiss Axio Observer Z1 fluorescence microscope with a 20× objective. Images were taken every 0.5 for 30 s and exposure time was 20 ms. Nondividing cells with visible posterior end in most frames were tracked for mean velocity analysis. The posterior end was manually tracked in Fiji.

### Immunoblot analysis

Samples lysed in Laemmli sample buffer were boiled at 100°C for 5 min. Proteins were resolved on SDS–polyacrylamide gel electrophoresis (SDS-PAGE) and transferred onto polyvinylidene difluoride (PVDF) membranes. Samples lysed in LDS (lithium dodecyl sulfate) sample buffer with dithiothreitol (DTT) were heated at 70°C for 10 min. Proteins were resolved on 3 to 8% tris-acetate gels and transferred onto PVDF membranes. Blots were firstly blocked with 3% BSA or non-fat milk in tris-buffered saline with 0.1% Tween-20 detergent (TBST) and incubated with the indicated primary antibodies followed by appropriate HRP-conjugated secondary antibodies. Chemiluminescent signal was detected and imaged using ImageQuant LAS 4000 mini (GE Healthcare). Membrane stripping, if required, was done in 0.1 M NaOH for 1 hour.

### Immunoprecipitation and silver staining

*T. brucei* cells (3 to 6 × 10^8^) were harvested and washed twice with PBS before cell lysis for 15 min on ice in 1 ml lysis buffer [20 mM tris-HCl (pH 7.5), 150 mM NaCl, 1 mM EDTA, and 0.5% NP-40] supplemented with 2× protease inhibitor (PI) cocktail. Cleared cell lysates were obtained by centrifugation at 17,000*g* for 15 min at 4°C. Supernatants were supplemented with 0.5 ml of binding buffer [10 mM tris-HCl (pH 7.5) and 150 mM NaCl] and then incubated with GFP-nanoantibodies magnetic agarose beads also known as GFP-Trap (Allele Biotech or Chromotek), EZview Red Anti-HA Affinity Gel (Millipore), or IgG Sepharose 6 Fast Flow beads (Cytiva) for 90 min at 4°C with rotation. The beads were washed three times in binding buffer containing 0.5× PI, eluted in Laemmli sample buffer, and fractionated by SDS-PAGE for further analyses by immunoblots or silver staining. Silver staining was performed according to Blum silver staining protocol (*72*). Protein bands of interest were excised for liquid chromatography tandem MS (LC-MS/MS) by the Mass Spectrometry Facility at Nanyang Technological University. For TbHYDIN-YFP immunoprecipitation, 3 × 10^9^ *T. brucei* cells were harvested and washed twice with PBS before cell lysis for 30 min on ice in 6 ml of high salt buffer (*73*) [50 mM Hepes (pH 7.4), 600 mM NaCl, 5 mM MgSO_4_, 0.5 mM EGTA, and 1 mM DTT] supplemented with 1% Triton X-100 and 2× PI. Additional 18 ml of 20 mM Hepes (pH 7.4) with 1× PI was added to reduce Triton X-100 concentration before centrifugation at 21,000*g* for 30 min. Supernatants were incubated with GFP-Trap (Chromotek) for 90 min at 4°C with rotation. The beads were washed three times in Hepes binding buffer [20 mM Hepes (pH 7.4) and 150 mM NaCl] containing 0.5× PI, eluted in LDS sample buffer with 100 mM DTT, and fractionated by tris-acetate gels immediately for further analyses by immunoblots.

HEK293T cells for immunoprecipitation using Anti-HA IP resin (GenScript) were lysed in 1 ml of lysis buffer supplemented with 2× PI for 30 min on ice. HEK293T cells for immunoprecipitation using GFP-Trap (Chromotek) were lysed in 1 ml of 1% NP-40 lysis buffer [20 mM tris-HCl (pH 7.5), 150 mM NaCl, 1 mM EDTA, and 1% NP-40] supplemented with 2× PI for 30 min on ice. Cleared lysates were obtained by centrifugation at 17,000*g* for 30 min at 4°C. NP-40 concentration was diluted to 0.5% with binding buffer. Beads were incubated with cleared cell lysates for 90 min at 4°C with rotation. The beads were washed three times in a wash buffer [10 mM tris-HCl (pH 7.5), 150 mM NaCl, and 0.05% NP-40] supplemented with 0.5× PI before eluted in Laemmli sample buffer for immunoblot analysis.

### Proximity-dependent biotin identification (BioID)

An optimized 5×GS linker was inserted between TbODA16 and 3×HA-BioID2 reporter to ensure correct cellular localization of the fusion protein. TbARL3A/TbARL3C dual RNAi was induced with tetracycline (10 μg/ml) for 24 hours before the induction of TbODA16-5×GS-3×HA-BioID2 expression with cumate (10 μg/ml) for an additional 24 hours. Biotin (50 μM) was then added to WT cells, TbODA16-5×GS-3×HA-BioID2 in cells with or without TbARL3A/TbARL3C dual RNAi for 24 hours. For each sample, 4 × 10^9^ cells were harvested and washed extensively with PBS to remove excess biotin. Cells were then lysed with 2 ml of BioID lysis buffer [1% SDS, 500 mM NaCl, 5 mM EDTA, 1 mM dithiothreitol, and 50 mM tris-HCl (pH 7.4)] supplemented with 2× PI for 15 min at room temperature. A total of 8 ml 1% NP-40 in PEM buffer (100 mM PIPES pH 6.9, 2 mM EGTA, and 1 mM MgSO_4_) with 2× PI was added to the mixture for another 15 min. After centrifugation (16,000*g*, 10 min, 16°C), the supernatant was incubated with 300 μl of Dynabeads M-280 Streptavidin (Invitrogen) for 4 hours at 4°C with rotation. Beads were washed once with PBS containing 0.5% SDS, twice with PBS containing 1% NP-40, and thrice with PBS. After washing, beads were subjected to disulfide reduction in 200 μl of triethylammonium (500 mM, pH 8.5) containing 4 mM tris(2-carboxyethyl)phosphine for 1 hour at 65°C with gentle agitation, followed by alkylation with addition of 4 μl of methyl methanethiosulfonate at room temperature for 15 min. Trypsin was added to 12.5 ng/μl for overnight digestion at 37°C. After trypsin digestion, the peptide solution was separated from beads, desalted, and analyzed by LC-MS/MS using a TripleTOF 5600 system. Proteomics data were analyzed on the ProteinPilot software 5.0 with 1% false discovery rate and searched against UniProt *T. brucei* proteome and common Repository of Adventitious Proteins. Exponentially modified protein abundance index (emPAI) (*74*) was calculated to assess protein abundance. Dot plots of selected proteins were generated by ProHits-viz (*75*).

### Transmission electron microscopy

*T. brucei* cells were firstly extracted with 1% NP-40 in PEME buffer [100 mM Pipes (pH 6.9), 1 mM MgSO_4_, 0.1 mM EDTA, and 2 mM EGTA] for 5 min at room temperature. Subsequently, isolated flagella were washed twice with PEME buffer and fixed with 1 ml of buffered fixative [2.5% glutaraldehyde, 2% paraformaldehyde, and 100 mM phosphate buffer (pH 7.0)]. Resin-embedded *T. brucei* samples were prepared according to established protocols (*76*). Embedded samples were subjected to ultrasectioning by a Leica Ultracut UCT Ultramicrotome to produce sections below 70 nm. After staining with UranyLess and Reynolds lead citrate (10 min each), these sections were imaged by Tecnai T12 (FEI).

### Cryo-electron tomography

*T. brucei* flagella were isolated following published protocols (*77*) with slight modifications. Cells were extracted in 1% NP-40 in PEME buffer supplemented with 1× PI and deoxyribonuclease I (0.25 mg/ml) for 10 min at room temperature and subsequent 30 min on ice. Extracted flagella were harvested by centrifugation at 16,000*g* for 10 min, washed twice with PEME buffer, and resuspended in the same buffer. Plasma-treated Quantifoil EM grids were mounted on a manual plunger, loaded with isolated flagellum suspension mixed with 10- or 15-nm gold fiducials (Electron Microscopy Sciences), blotted from the back side using Whatman paper #5, and plunged into ethane precooled to liquid nitrogen temperature. Isolated flagella were imaged using a 300-kV Titan Krios electron microscope (Thermo Fisher Scientific) equipped with an energy filter (Gatan), a K3 direct electron detector (Gatan), and an objective aperture or a Volta phase plate (*78*). High-magnification images were recorded at ×42,000 and ×33,000, respectively, corresponding to a pixel size of 2.2 or 2.7 Å. Tilt series were recorded using Tomo4 software with bidirectional acquisition schemes (*79*), each from −50° to 50° with 2° increment. Target defocus was set to −4 to −8 μm. The total dose was limited to 90 to 100 e/Å^2^. Upon phase plate alignment and conditioning, tilt series of the flagellum were recorded at ×33,000 at pixel size 2.7 Å using Tomo4 software with bidirectional acquisition schemes, each from −50° to 50° with 2° increment. Target defocus was set to −1.0 μm. Every two or three tilt series, a new spot on the phase plate was selected. The total dose was limited to 110 e/Å^2^. Tilt series alignment and tomogram reconstruction are performed automatically using tomography pipeline described in (*80*). Subcellular features were semi-automatically segmented using EMAN2 and refined manually using Chimera (*81*) or ChimeraX (*82*, *83*).

### Bioinformatic analysis

All *T. brucei* gene sequences were retrieved from TriTrypDB.org (*84*). Sequence information and domain prediction were retrieved from UniProt (*85*). The schematic diagram of domains was achieved with Illustrator for Biological Sequences (IBS) web server (*86*). Sequences of ODA16 homologs were aligned with MUSCLE (*87*) and visualized with ESPript (*88*). Secondary structures of HsODA16 (Protein Data Bank: 5NNZ) were attached to the alignment for reference.

### Image processing and statistical analysis

Fiji software (*89*) with appropriate plugins was used for image processing. Intensity and length measurements were performed on *T. brucei* cells in early cell cycle stages with a single flagellum. Statistical analysis and graphing were carried out on GraphPad. Sample size (*N*) and applied analysis were included in corresponding figure legends. All data were presented as means ± SD. Statistical analysis for comparing two groups was performed using unpaired *t* test with Welch’s correction. Statistical differences among multiple experimental groups were assessed using one-way analysis of variance (ANOVA) with Tukey’s multiple comparisons test. *P* values < 0.05 are considered significantly different. All experiments except for BioID MS analysis have at least two independent biological replicates.

## References

[1] J. F. Reiter, M. R. Leroux, Genes and molecular pathways underpinning ciliopathies. Nat. Rev. Mol. Cell Biol. 18, 533–547 (2017). 28698599 10.1038/nrm.2017.60 PMC5851292

[2] T. Ishikawa, Axoneme structure from motile cilia. Cold Spring Harb. Perspect. Biol. 9, a028076 (2017). 27601632 10.1101/cshperspect.a028076 PMC5204319

[3] J. L. Rosenbaum, G. B. Witman, Intraflagellar transport. Nat. Rev. Mol. Cell Biol. 3, 813–825 (2002). 12415299 10.1038/nrm952

[4] J. M. Scholey, Intraflagellar transport. Annu. Rev. Cell Dev. Biol. 19, 423–443 (2003). 14570576 10.1146/annurev.cellbio.19.111401.091318

[5] S. Bhogaraju, L. Cajanek, C. Fort, T. Blisnick, K. Weber, M. Taschner, N. Mizuno, S. Lamla, P. Bastin, E. A. Nigg, E. Lorentzen, Molecular basis of tubulin transport within the cilium by IFT74 and IFT81. Science 341, 1009–1012 (2013). 23990561 10.1126/science.1240985 PMC4359902

[6] K. Lechtreck, Cargo adapters expand the transport range of intraflagellar transport. J. Cell Sci. 135, jcs260408 (2022). 36533425 10.1242/jcs.260408 PMC9845741

[7] N. T. Ahmed, C. Gao, B. F. Lucker, D. G. Cole, D. R. Mitchell, ODA16 aids axonemal outer row dynein assembly through an interaction with the intraflagellar transport machinery. J. Cell Biol. 183, 313–322 (2008). 18852297 10.1083/jcb.200802025 PMC2568026

[8] E. L. Hunter, K. Lechtreck, G. Fu, J. Hwang, H. Lin, A. Gokhale, L. M. Alford, B. Lewis, R. Yamamoto, R. Kamiya, F. Yang, D. Nicastro, S. K. Dutcher, M. Wirschell, W. S. Sale, The IDA3 adapter, required for intraflagellar transport of I1 dynein, is regulated by ciliary length. Mol. Biol. Cell 29, 886–896 (2018). 29467251 10.1091/mbc.E17-12-0729 PMC5896928

[9] H. Jin, S. R. White, T. Shida, S. Schulz, M. Aguiar, S. P. Gygi, J. F. Bazan, M. V. Nachury, The conserved Bardet-Biedl syndrome proteins assemble a coat that traffics membrane proteins to cilia. Cell 141, 1208–1219 (2010). 20603001 10.1016/j.cell.2010.05.015 PMC2898735

[10] P. Liu, K. F. Lechtreck, The Bardet-Biedl syndrome protein complex is an adapter expanding the cargo range of intraflagellar transport trains for ciliary export. Proc. Natl. Acad. Sci. U.S.A. 115, E934–E943 (2018). 29339469 10.1073/pnas.1713226115 PMC5798339

[11] K. N. Wren, J. M. Craft, D. Tritschler, A. Schauer, D. K. Patel, E. F. Smith, M. E. Porter, P. Kner, K. F. Lechtreck, A differential cargo-loading model of ciliary length regulation by IFT. Curr. Biol. 23, 2463–2471 (2013). 24316207 10.1016/j.cub.2013.10.044 PMC3881561

[12] G. Pigino, Intraflagellar transport. Curr. Biol. 31, R530–R536 (2021). 34033785 10.1016/j.cub.2021.03.081

[13] C.-H. Sung, M. R. Leroux, The roles of evolutionarily conserved functional modules in cilia-related trafficking. Nat. Cell Biol. 15, 1387–1397 (2013). 24296415 10.1038/ncb2888 PMC4016715

[14] T. Caspary, C. E. Larkins, K. V. Anderson, The graded response to Sonic Hedgehog depends on cilia architecture. Dev. Cell 12, 767–778 (2007). 17488627 10.1016/j.devcel.2007.03.004

[15] M. C. Humbert, K. Weihbrecht, C. C. Searby, Y. Li, R. M. Pope, V. C. Sheffield, S. Seo, ARL13B, PDE6D, and CEP164 form a functional network for INPP5E ciliary targeting. Proc. Natl. Acad. Sci. U.S.A. 109, 19691–19696 (2012). 23150559 10.1073/pnas.1210916109 PMC3511769

[16] C. Hanke-Gogokhia, Z. Wu, C. D. Gerstner, J. M. Frederick, H. Zhang, W. Baehr, Arf-like protein 3 (ARL3) regulates protein trafficking and ciliogenesis in mouse photoreceptors. J. Biol. Chem. 291, 7142–7155 (2016). 26814127 10.1074/jbc.M115.710954 PMC4807295

[17] Y. Li, Q. Wei, Y. Zhang, K. Ling, J. Hu, The small GTPases ARL-13 and ARL-3 coordinate intraflagellar transport and ciliogenesis. J. Cell Biol. 189, 1039–1051 (2010). 20530210 10.1083/jcb.200912001 PMC2886347

[18] S. A. Ismail, Y.-X. Chen, M. Miertzschke, I. R. Vetter, C. Koerner, A. Wittinghofer, Structural basis for Arl3-specific release of myristoylated ciliary cargo from UNC119. EMBO J. 31, 4085–4094 (2012). 22960633 10.1038/emboj.2012.257 PMC3474929

[19] S. A. Ismail, Y.-X. Chen, A. Rusinova, A. Chandra, M. Bierbaum, L. Gremer, G. Triola, H. Waldmann, P. I. Bastiaens, A. Wittinghofer, Arl2-GTP and Arl3-GTP regulate a GDI-like transport system for farnesylated cargo. Nat. Chem. Biol. 7, 942–949 (2011). 22002721 10.1038/nchembio.686

[20] K. Gotthardt, M. Lokaj, C. Koerner, N. Falk, A. Gießl, A. Wittinghofer, A G-protein activation cascade from Arl13B to Arl3 and implications for ciliary targeting of lipidated proteins. eLife 4, e11859 (2015). 26551564 10.7554/eLife.11859 PMC4868535

[21] V. L. Jensen, M. R. Leroux, Gates for soluble and membrane proteins, and two trafficking systems (IFT and LIFT), establish a dynamic ciliary signaling compartment. Curr. Opin. Cell Biol. 47, 83–91 (2017). 28432921 10.1016/j.ceb.2017.03.012

[22] Y.-X. Liu, W.-Y. Sun, B. Xue, R.-K. Zhang, W.-J. Li, X. Xie, Z.-C. Fan, ARL3 mediates BBSome ciliary turnover by promoting its outward movement across the transition zone. J. Cell Biol. 221, e202111076 (2022). 36129685 10.1083/jcb.202111076 PMC9499826

[23] J. Dai, G. Zhang, R. A. Alkhofash, B. Mekonnen, S. Saravanan, B. Xue, Z.-C. Fan, E. Betleja, D. G. Cole, P. Liu, K. Lechtreck, Loss of ARL13 impedes BBSome-dependent cargo export from *Chlamydomonas* cilia. J. Cell Biol. 221, e202201050 (2022). 36040375 10.1083/jcb.202201050 PMC9436004

[24] A. Cuvillier, F. Redon, J. C. Antoine, P. Chardin, T. DeVos, G. Merlin, LdARL-3A, a Leishmania promastigote-specific ADP-ribosylation factor-like protein, is essential for flagellum integrity. J. Cell Sci. 113, 2065–2074 (2000). 10806117 10.1242/jcs.113.11.2065

[25] Y. Zhang, Y. Huang, A. Srivathsan, T. K. Lim, Q. Lin, C. Y. He, The unusual flagellar-targeting mechanism and functions of the trypanosome ortholog of the ciliary GTPase Arl13b. J. Cell Sci. 131, jcs219071 (2018). 30097558 10.1242/jcs.219071 PMC6140319

[26] D. Steverding, The history of African trypanosomiasis. Parasit. Vectors 1, 3 (2008). 18275594 10.1186/1756-3305-1-3 PMC2270819

[27] M. Pandey, Y. Huang, T. K. Lim, Q. Lin, C. Y. He, Flagellar targeting of an arginine kinase requires a conserved lipidated protein intraflagellar transport (LIFT) pathway in *Trypanosoma brucei*. J. Biol. Chem. 295, 11326–11336 (2020). 32587088 10.1074/jbc.RA120.014287 PMC7415996

[28] S. Ohshima, M. Ohashi-Suzuki, Y. Miura, Y. Yabu, N. Okada, N. Ohta, T. Suzuki, TbUNC119 and its binding protein complex are essential for propagation, motility, and morphogenesis of Trypanosoma brucei procyclic form cells. PLOS ONE 5, e15577 (2010). 21203515 10.1371/journal.pone.0015577 PMC3008729

[29] F. J. Li, Z. S. Xu, H. M. Aye, A. Brasseur, Z. R. Lun, K. S. W. Tan, C. Y. He, An efficient cumate-inducible system for procyclic and bloodstream form *Trypanosoma brucei*. Mol. Biochem. Parasitol. 214, 101–104 (2017). 28438458 10.1016/j.molbiopara.2017.04.007

[30] H. P. Price, A. Peltan, M. Stark, D. F. Smith, The small GTPase ARL2 is required for cytokinesis in *Trypanosoma brucei*. Mol. Biochem. Parasitol. 173, 123–131 (2010). 20653091 10.1016/j.molbiopara.2010.05.016 PMC2913242

[31] M. Taschner, A. Mourão, M. Awasthi, J. Basquin, E. Lorentzen, Structural basis of outer dynein arm intraflagellar transport by the transport adaptor protein ODA16 and the intraflagellar transport protein IFT46. J. Biol. Chem. 292, 7462–7473 (2017). 28298440 10.1074/jbc.M117.780155 PMC5418046

[32] Y. Hou, G. B. Witman, The N-terminus of IFT46 mediates intraflagellar transport of outer arm dynein and its cargo-adaptor ODA16. Mol. Biol. Cell 28, 2420–2433 (2017). 28701346 10.1091/mbc.E17-03-0172 PMC5576905

[33] P. B. Desai, A. B. Dean, D. R. Mitchell, “4 - Cytoplasmic preassembly and trafficking of axonemal dyneins,” in *Dyneins: Structure, Biology and Disease (Second Edition),* S. M. King, Ed. (Academic Press, 2018), pp. 140–161.

[34] J. Dai, F. Barbieri, D. R. Mitchell, K. F. Lechtreck, In vivo analysis of outer arm dynein transport reveals cargo-specific intraflagellar transport properties. Mol. Biol. Cell 29, 2553–2565 (2018). 30133350 10.1091/mbc.E18-05-0291 PMC6254574

[35] Y. Hou, H. Qin, J. A. Follit, G. J. Pazour, J. L. Rosenbaum, G. B. Witman, Functional analysis of an individual IFT protein: IFT46 is required for transport of outer dynein arms into flagella. J. Cell Biol. 176, 653–665 (2007). 17312020 10.1083/jcb.200608041 PMC2064023

[36] H. Q. Dang, Q. Zhou, V. W. Rowlett, H. Hu, K. J. Lee, W. Margolin, Z. Li, Proximity interactions among basal body components in *Trypanosoma brucei* identify novel regulators of basal body biogenesis and inheritance. mBio 8, e02120-16 (2017). 28049148 10.1128/mBio.02120-16 PMC5210500

[37] C. Branche, L. Kohl, G. Toutirais, J. Buisson, J. Cosson, P. Bastin, Conserved and specific functions of axoneme components in trypanosome motility. J. Cell Sci. 119, 3443–3455 (2006). 16882690 10.1242/jcs.03078

[38] B. Wickstead, K. Gull, Dyneins across eukaryotes: A comparative genomic analysis. Traffic 8, 1708–1721 (2007). 17897317 10.1111/j.1600-0854.2007.00646.x PMC2239267

[39] E. A. Bearce, Z. H. Irons, S. B. Craig, C. J. Kuhns, C. Sabazali, D. R. Farnsworth, A. C. Miller, D. T. Grimes, Daw1 regulates the timely onset of cilia motility during development. Development 149, dev200017 (2022). 35708608 10.1242/dev.200017 PMC9270974

[40] J. S. Leslie, R. Hjeij, A. Vivante, E. A. Bearce, L. Dyer, J. Wang, L. Rawlins, J. Kennedy, N. Ubeyratna, J. Fasham, Z. H. Irons, S. B. Craig, J. Koenig, S. George, B. Pode-Shakked, Y. Bolkier, O. Barel, S. Mane, K. K. Frederiksen, O. Wenger, E. Scott, H. E. Cross, E. Lorentzen, D. P. Norris, Y. Anikster, H. Omran, D. T. Grimes, A. H. Crosby, E. L. Baple, Biallelic DAW1 variants cause a motile ciliopathy characterized by laterality defects and subtle ciliary beating abnormalities. Genet. Med 24, 2249–2261 (2022). 36074124 10.1016/j.gim.2022.07.019 PMC10584193

[41] H. R. Dawe, M. K. Shaw, H. Farr, K. Gull, The hydrocephalus inducing gene product, Hydin, positions axonemal central pair microtubules. BMC Biol. 5, 33 (2007). 17683645 10.1186/1741-7007-5-33 PMC2048497

[42] D. R. Mitchell, Orientation of the central pair complex during flagellar bend formation in Chlamydomonas. Cell Motil. Cytoskeleton 56, 120–129 (2003). 14506709 10.1002/cm.10142

[43] C. K. Omoto, C. Kung, The pair of central tubules rotates during ciliary beat in Paramecium. Nature 279, 532–534 (1979). 450097 10.1038/279532a0

[44] N. T. Ahmed, D. R. Mitchell, ODA16p, a Chlamydomonas flagellar protein needed for dynein assembly. Mol. Biol. Cell 16, 5004–5012 (2005). 16093345 10.1091/mbc.E05-07-0627 PMC1237099

[45] E. K. Fansa, A. Wittinghofer, Sorting of lipidated cargo by the Arl2/Arl3 system. Small GTPases 7, 222–230 (2016). 27806215 10.1080/21541248.2016.1224454 PMC5129900

[46] J. Wang, M. Taschner, N. A. Petriman, M. B. Andersen, J. Basquin, S. Bhogaraju, M. Vetter, S. Wachter, A. Lorentzen, E. Lorentzen, Purification and crystal structure of human ODA16: Implications for ciliary import of outer dynein arms by the intraflagellar transport machinery. Protein Sci. 29, 1502–1510 (2020). 32239748 10.1002/pro.3864 PMC7255517

[47] S. C. Jin, J. Homsy, S. Zaidi, Q. Lu, S. Morton, S. R. DePalma, X. Zeng, H. Qi, W. Chang, M. C. Sierant, W.-C. Hung, S. Haider, J. Zhang, J. Knight, R. D. Bjornson, C. Castaldi, I. R. Tikhonoa, K. Bilguvar, S. M. Mane, S. J. Sanders, S. Mital, M. W. Russell, J. W. Gaynor, J. Deanfield, A. Giardini, G. A. Porter Jr., D. Srivastava, C. W. Lo, Y. Shen, W. S. Watkins, M. Yandell, H. J. Yost, M. Tristani-Firouzi, J. W. Newburger, A. E. Roberts, R. Kim, H. Zhao, J. R. Kaltman, E. Goldmuntz, W. K. Chung, J. G. Seidman, B. D. Gelb, C. E. Seidman, R. P. Lifton, M. Brueckner, Contribution of rare inherited and de novo variants in 2,871 congenital heart disease probands. Nat. Genet. 49, 1593–1601 (2017). 28991257 10.1038/ng.3970 PMC5675000

[48] B. Braschi, H. Omran, G. B. Witman, G. J. Pazour, K. K. Pfister, E. A. Bruford, S. M. King, Consensus nomenclature for dyneins and associated assembly factors. J. Cell Biol. 221, e202109014 (2022). 35006274 10.1083/jcb.202109014 PMC8754002

[49] A. Kobayashi, S. Kubota, N. Mori, M. J. McLaren, G. Inana, Photoreceptor synaptic protein HRG4 (UNC119) interacts with ARL2 via a putative conserved domain. FEBS Lett. 534, 26–32 (2003). 12527357 10.1016/s0014-5793(02)03766-3

[50] K. J. Wright, L. M. Baye, A. Olivier-Mason, S. Mukhopadhyay, L. Sang, M. Kwong, W. Wang, P. R. Pretorius, V. C. Sheffield, P. Sengupta, D. C. Slusarski, P. K. Jackson, An ARL3-UNC119-RP2 GTPase cycle targets myristoylated NPHP3 to the primary cilium. Genes Dev. 25, 2347–2360 (2011). 22085962 10.1101/gad.173443.111 PMC3222901

[51] A. A. Ivanova, M. P. East, S. L. Yi, R. A. Kahn, Characterization of recombinant ELMOD (cell engulfment and motility domain) proteins as GTPase-activating proteins (GAPs) for ARF family GTPases. J. Biol. Chem. 289, 11111–11121 (2014). 24616099 10.1074/jbc.M114.548529 PMC4036250

[52] Y. ElMaghloob, B. Sot, M. J. McIlwraith, E. Garcia, T. Yelland, S. Ismail, ARL3 activation requires the co-GEF BART and effector-mediated turnover. eLife 10, e64624 (2021). 33438581 10.7554/eLife.64624 PMC7817177

[53] Z. C. Wright, Y. Loskutov, D. Murphy, P. Stoilov, E. Pugacheva, A. F. X. Goldberg, V. Ramamurthy, ADP-ribosylation factor-like 2 (ARL2) regulates cilia stability and development of outer segments in rod photoreceptor neurons. Sci. Rep. 8, 16967 (2018). 30446707 10.1038/s41598-018-35395-3 PMC6240099

[54] A. R. Moye, R. Singh, V. A. Kimler, T. L. Dilan, D. Munezero, T. Saravanan, A. F. X. Goldberg, V. Ramamurthy, ARL2BP, a protein linked to retinitis pigmentosa, is needed for normal photoreceptor cilia doublets and outer segment structure. Mol. Biol. Cell 29, 1590–1598 (2018). 29718757 10.1091/mbc.E18-01-0040 PMC6080659

[55] A. R. Moye, N. Bedoni, J. G. Cunningham, U. Sanzhaeva, E. S. Tucker, P. Mathers, V. G. Peter, M. Quinodoz, L. P. Paris, L. Coutinho-Santos, P. Camacho, M. G. Purcell, A. C. Winkelmann, J. A. Foster, E. N. Pugacheva, C. Rivolta, V. Ramamurthy, Mutations in ARL2BP, a protein required for ciliary microtubule structure, cause syndromic male infertility in humans and mice. PLOS Genet. 15, e1008315 (2019). 31425546 10.1371/journal.pgen.1008315 PMC6715254

[56] G. R. Mali, F. A. Ali, C. K. Lau, F. Begum, J. Boulanger, J. D. Howe, Z. A. Chen, J. Rappsilber, M. Skehel, A. P. Carter, Shulin packages axonemal outer dynein arms for ciliary targeting. Science 371, 910–916 (2021). 33632841 10.1126/science.abe0526 PMC7116892

[57] G. M. Solomon, R. Francis, K. K. Chu, S. E. Birket, G. Gabriel, J. E. Trombley, K. L. Lemke, N. Klena, B. Turner, G. J. Tearney, C. W. Lo, S. M. Rowe, Assessment of ciliary phenotype in primary ciliary dyskinesia by micro-optical coherence tomography. JCI Insight 2, e91702 (2017). 28289722 10.1172/jci.insight.91702 PMC5333960

[58] Y. Li, N. T. Klena, G. C. Gabriel, X. Liu, A. J. Kim, K. Lemke, Y. Chen, B. Chatterjee, W. Devine, R. R. Damerla, C. Chang, H. Yagi, J. T. San Agustin, M. Thahir, S. Anderton, C. Lawhead, A. Vescovi, H. Pratt, J. Morgan, L. Haynes, C. L. Smith, J. T. Eppig, L. Reinholdt, R. Francis, L. Leatherbury, M. K. Ganapathiraju, K. Tobita, G. J. Pazour, C. W. Lo, Global genetic analysis in mice unveils central role for cilia in congenital heart disease. Nature 521, 520–524 (2015). 25807483 10.1038/nature14269 PMC4617540

[59] I. Cunningham, New culture medium for maintenance of tsetse tissues and growth of trypanosomatids. J. Protozool. 24, 325–329 (1977). 881656 10.1111/j.1550-7408.1977.tb00987.x

[60] L. Ruben, C. Egwuagu, C. L. Patton, African trypanosomes contain calmodulin which is distinct from host calmodulin. Biochim. Biophys. Acta 758, 104–113 (1983). 6135450 10.1016/0304-4165(83)90290-8

[61] E. Wirtz, S. Leal, C. Ochatt, G. A. Cross, A tightly regulated inducible expression system for conditional gene knock-outs and dominant-negative genetics in Trypanosoma brucei. Mol. Biochem. Parasitol. 99, 89–101 (1999). 10215027 10.1016/s0166-6851(99)00002-x

[62] M. J. van den Hoff, A. F. Moorman, W. H. Lamers, Electroporation in ‘intracellular’ buffer increases cell survival. Nucleic Acids Res. 20, 2902 (1992). 1614888 10.1093/nar/20.11.2902 PMC336954

[63] D. I. Kim, S. C. Jensen, K. A. Noble, B. Kc, K. H. Roux, K. Motamedchaboki, K. J. Roux, An improved smaller biotin ligase for BioID proximity labeling. Mol. Biol. Cell 27, 1188–1196 (2016). 26912792 10.1091/mbc.E15-12-0844 PMC4831873

[64] J. Y. Ong, Synonymous mutation generator: A web tool for designing RNAi-resistant sequences. bioRxiv 425100 [**Preprint**]. 17 March 2021.

[65] S. Redmond, J. Vadivelu, M. C. Field, RNAit: An automated web-based tool for the selection of RNAi targets in *Trypanosoma brucei*. Mol. Biochem. Parasitol. 128, 115–118 (2003). 12706807 10.1016/s0166-6851(03)00045-8

[66] B. Wickstead, K. Ersfeld, K. Gull, Targeting of a tetracycline-inducible expression system to the transcriptionally silent minichromosomes of *Trypanosoma brucei*. Mol. Biochem. Parasitol. 125, 211–216 (2002). 12467990 10.1016/s0166-6851(02)00238-4

[67] S. Dean, J. Sunter, R. J. Wheeler, I. Hodkinson, E. Gluenz, K. Gull, A toolkit enabling efficient, scalable and reproducible gene tagging in trypanosomatids. Open Biol. 5, 140197 (2015). 25567099 10.1098/rsob.140197 PMC4313374

[68] M. Oberholzer, S. Morand, S. Kunz, T. Seebeck, A vector series for rapid PCR-mediated C-terminal in situ tagging of Trypanosoma brucei genes. Mol. Biochem. Parasitol. 145, 117–120 (2006). 16269191 10.1016/j.molbiopara.2005.09.002

[69] J. D. Bangs, E. M. Brouch, D. M. Ransom, J. L. Roggy, A soluble secretory reporter system in *Trypanosoma brucei*. Studies on endoplasmic reticulum targeting. J. Biol. Chem. 271, 18387–18393 (1996). 8702482 10.1074/jbc.271.31.18387

[70] T. T. Yang, M. N. T. Tran, W. M. Chong, C. E. Huang, J. C. Liao, Single-particle tracking localization microscopy reveals nonaxonemal dynamics of intraflagellar transport proteins at the base of mammalian primary cilia. Mol. Biol. Cell 30, 828–837 (2019). 30759057 10.1091/mbc.E18-10-0654 PMC6589787

[71] P. Bastin, Z. Bagherzadeh, K. R. Matthews, K. Gull, A novel epitope tag system to study protein targeting and organelle biogenesis in *Trypanosoma brucei*. Mol. Biochem. Parasitol. 77, 235–239 (1996). 8813669 10.1016/0166-6851(96)02598-4

[72] E. Mortz, T. N. Krogh, H. Vorum, A. Gorg, Improved silver staining protocols for high sensitivity protein identification using matrix-assisted laser desorption/ionization-time of flight analysis. Proteomics 1, 1359–1363 (2001). 11922595 10.1002/1615-9861(200111)1:11<1359::AID-PROT1359>3.0.CO;2-Q

[73] Q. Rao, L. Han, Y. Wang, P. Chai, Y. W. Kuo, R. Yang, F. Hu, Y. Yang, J. Howard, K. Zhang, Structures of outer-arm dynein array on microtubule doublet reveal a motor coordination mechanism. Nat. Struct. Mol. Biol. 28, 799–810 (2021). 34556869 10.1038/s41594-021-00656-9 PMC8500839

[74] Y. Ishihama, Y. Oda, T. Tabata, T. Sato, T. Nagasu, J. Rappsilber, M. Mann, Exponentially modified protein abundance index (emPAI) for estimation of absolute protein amount in proteomics by the number of sequenced peptides per protein. Mol. Cell. Proteomics 4, 1265–1272 (2005). 15958392 10.1074/mcp.M500061-MCP200

[75] J. D. R. Knight, H. Choi, G. D. Gupta, L. Pelletier, B. Raught, A. I. Nesvizhskii, A. C. Gingras, ProHits-viz: A suite of web tools for visualizing interaction proteomics data. Nat. Methods 14, 645–646 (2017). 28661499 10.1038/nmeth.4330 PMC5831326

[76] J. L. Hoog, E. Gluenz, S. Vaughan, K. Gull, Ultrastructural investigation methods for *Trypanosoma brucei*. Methods Cell Biol. 96, 175–196 (2010). 20869523 10.1016/S0091-679X(10)96008-1

[77] L. C. Hughes, K. S. Ralston, K. L. Hill, Z. H. Zhou, Three-dimensional structure of the Trypanosome flagellum suggests that the paraflagellar rod functions as a biomechanical spring. PLOS ONE 7, e25700 (2012). 22235240 10.1371/journal.pone.0025700 PMC3250385

[78] R. Danev, B. Buijsse, M. Khoshouei, J. M. Plitzko, W. Baumeister, Volta potential phase plate for in-focus phase contrast transmission electron microscopy. Proc. Natl. Acad. Sci. U.S.A. 111, 15635–15640 (2014). 25331897 10.1073/pnas.1418377111 PMC4226124

[79] Q. S. Zheng, M. B. Braunfeld, J. W. Sedat, D. A. Agard, An improved strategy for automated electron microscopic tomography. J. Struct. Biol. 147, 91–101 (2004). 15193638 10.1016/j.jsb.2004.02.005

[80] M. Chen, J. M. Bell, X. Shi, S. Y. Sun, Z. Wang, S. J. Ludtke, A complete data processing workflow for cryo-ET and subtomogram averaging. Nat. Methods 16, 1161–1168 (2019). 31611690 10.1038/s41592-019-0591-8 PMC6858567

[81] E. F. Pettersen, T. D. Goddard, C. C. Huang, G. S. Couch, D. M. Greenblatt, E. C. Meng, T. E. Ferrin, UCSF Chimera—A visualization system for exploratory research and analysis. J. Comput. Chem. 25, 1605–1612 (2004). 15264254 10.1002/jcc.20084

[82] T. D. Goddard, C. C. Huang, E. C. Meng, E. F. Pettersen, G. S. Couch, J. H. Morris, T. E. Ferrin, UCSF ChimeraX: Meeting modern challenges in visualization and analysis. Protein Sci. 27, 14–25 (2018). 28710774 10.1002/pro.3235 PMC5734306

[83] E. F. Pettersen, T. D. Goddard, C. C. Huang, E. C. Meng, G. S. Couch, T. I. Croll, J. H. Morris, T. E. Ferrin, UCSF ChimeraX: Structure visualization for researchers, educators, and developers. Protein Sci. 30, 70–82 (2021). 32881101 10.1002/pro.3943 PMC7737788

[84] A. Shanmugasundram, D. Starns, U. Bohme, B. Amos, P. A. Wilkinson, O. S. Harb, S. Warrenfeltz, J. C. Kissinger, M. A. McDowell, D. S. Roos, K. Crouch, A. R. Jones, TriTrypDB: An integrated functional genomics resource for kinetoplastida. PLOS Negl. Trop. Dis. 17, e0011058 (2023). 36656904 10.1371/journal.pntd.0011058 PMC9888696

[85] C. UniProt, UniProt: The universal protein knowledgebase in 2023. Nucleic Acids Res. 51, D523–D531 (2023). 36408920 10.1093/nar/gkac1052 PMC9825514

[86] W. Liu, Y. Xie, J. Ma, X. Luo, P. Nie, Z. Zuo, U. Lahrmann, Q. Zhao, Y. Zheng, Y. Zhao, Y. Xue, J. Ren, IBS: An illustrator for the presentation and visualization of biological sequences. Bioinformatics 31, 3359-3361 (2015). 26069263 10.1093/bioinformatics/btv362 PMC4595897

[87] F. Madeira, Y. M. Park, J. Lee, N. Buso, T. Gur, N. Madhusoodanan, P. Basutkar, A. R. N. Tivey, S. C. Potter, R. D. Finn, R. Lopez, The EMBL-EBI search and sequence analysis tools APIs in 2019. Nucleic Acids Res. 47, W636–W641 (2019). 30976793 10.1093/nar/gkz268 PMC6602479

[88] X. Robert, P. Gouet, Deciphering key features in protein structures with the new ENDscript server. Nucleic Acids Res. 42, W320–W324 (2014). 24753421 10.1093/nar/gku316 PMC4086106

[89] J. Schindelin, I. Arganda-Carreras, E. Frise, V. Kaynig, M. Longair, T. Pietzsch, S. Preibisch, C. Rueden, S. Saalfeld, B. Schmid, J. Y. Tinevez, D. J. White, V. Hartenstein, K. Eliceiri, P. Tomancak, A. Cardona, Fiji: an open-source platform for biological-image analysis. Nat. Methods 9, 676–682 (2012). 22743772 10.1038/nmeth.2019 PMC3855844

[90] S. Veltel, R. Gasper, E. Eisenacher, A. Wittinghofer, The retinitis pigmentosa 2 gene product is a GTPase-activating protein for Arf-like 3. Nat. Struct. Mol. Biol. 15, 373–380 (2008). 18376416 10.1038/nsmb.1396

[91] Y. Perez-Riverol, J. Bai, C. Bandla, D. Garcia-Seisdedos, S. Hewapathirana, S. Kamatchinathan, D. J. Kundu, A. Prakash, A. Frericks-Zipper, M. Eisenacher, M. Walzer, S. Wang, A. Brazma, J. A. Vizcaino, The PRIDE database resources in 2022: A hub for mass spectrometry-based proteomics evidences. Nucleic Acids Res. 50, D543–D552 (2022). 34723319 10.1093/nar/gkab1038 PMC8728295

