# Supplementary material for: ARL3 GTPases facilitate ODA16 unloading from IFT in motile cilia
Source: Sci Adv. 2024 Sep 4;10(36):eadq2950. doi: 10.1126/sciadv.adq2950 (PMC11373600; doi:10.1126/sciadv.adq2950)
Supplement: Supplementary file 1 — Figs. S1 to S7 Table S1 Legends for movies S1 to S3 [file sciadv.adq2950_sm.pdf]

Science Advances
AAAS

Supplementary Materials for

ARL3 GTPases facilitate ODA16 unloading from IFT in motile cilia

Yameng Huang *et al.*

Corresponding author: Cynthia Y. He, dbshyc@nus.edu.sg

Sci. Adv. **10**, eadq2950 (2024)
DOI: 10.1126/sciadv.adq2950

The PDF file includes:

Figs. S1 to S7
Table S1
Legends for movies S1 to S3

Other Supplementary Material for this manuscript includes the following:

Movies S1 to S3

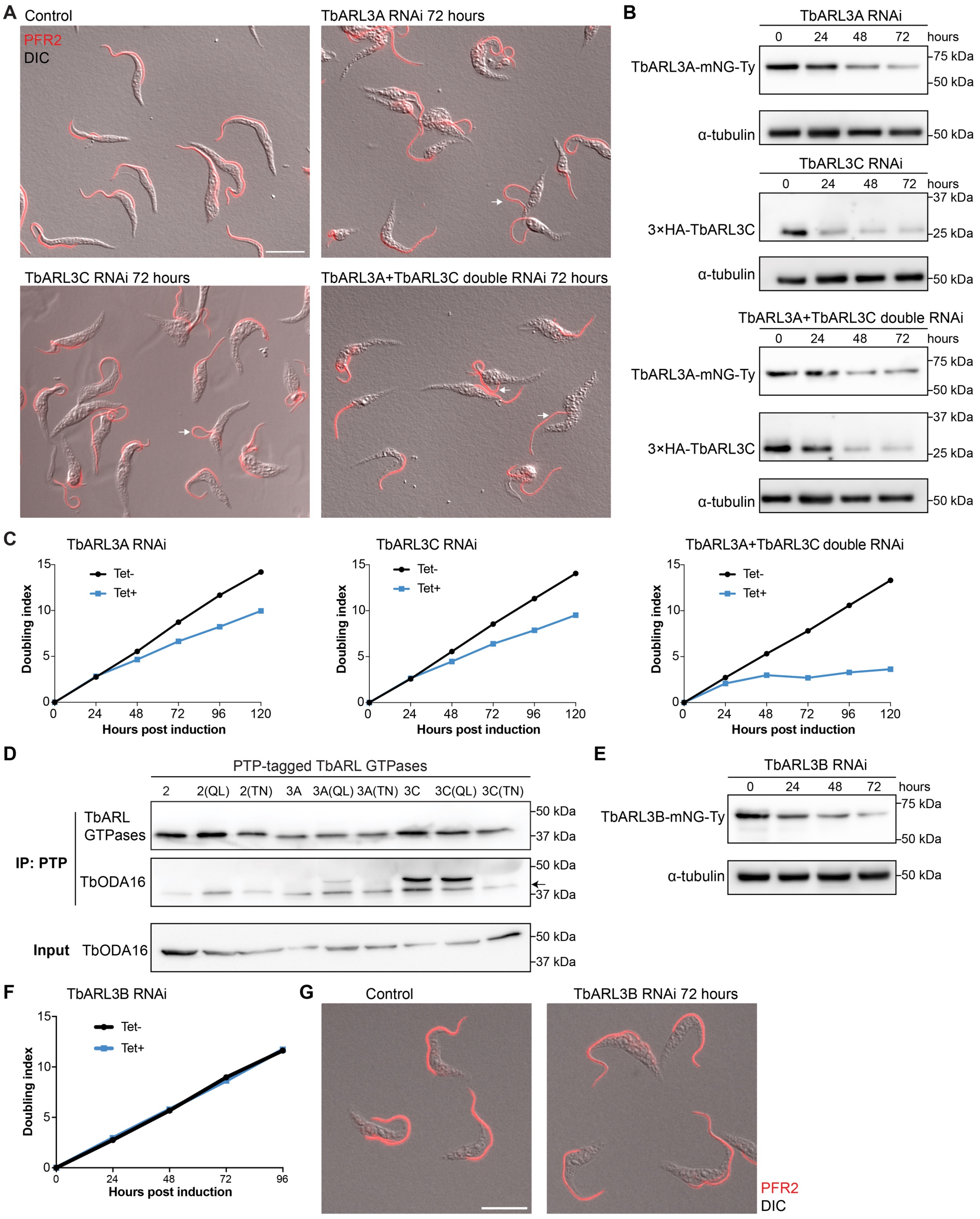

Fig. S1. TbARL3A and TbARL3C are synthetically lethal.

(**A**) *T. brucei* cells were stably transfected with tetracycline (Tet)-inducible RNAi of TbARL3A and TbARL3C, individually or together. In control cells, the flagellum is laterally attached to the

cell body. In all RNAi cells, morphology of the flagellum and its attachment to the cell body were affected. Regions of flagellar detachment from the cell body were marked by arrows. Scale bar: 10 μm.
(**B**) Efficient depletion of TbARL3A and/or TbARL3C in the RNAi cells was confirmed by immunoblots of endogenously tagged TbARL3A-mNeonGreen (mNG)-Ty and 3×HA-TbARL3C with anti-Ty and anti-HA antibodies. α-tubulin was used as loading control.
(**C**) Growth assays of TbARL3A RNAi, TbARL3C RNAi and dual RNAi cells, in the absence or presence of tetracycline for RNAi induction.
(**D**) Interaction of TbODA16 with TbARL2 and TbARL3 variants by co-IP assays using IgG Sepharose 6 Fast Flow beads. The anti-TbODA16 antibodies cross-reacted with the PTP tag and therefore also labeled the PTP-tagged TbARL GTPases at ~40 kDa (arrow).
(**E**) Immunoblots showing depletion of TbARL3B by inducible RNAi with 10 μg/mL tetracycline.
(**F**) Growth assays of TbARL3B RNAi cells.
(**G**) Immunofluorescence of cells non-induced or induced for TbARL3B RNAi for 72 hours. Scale bar: 10 μm.

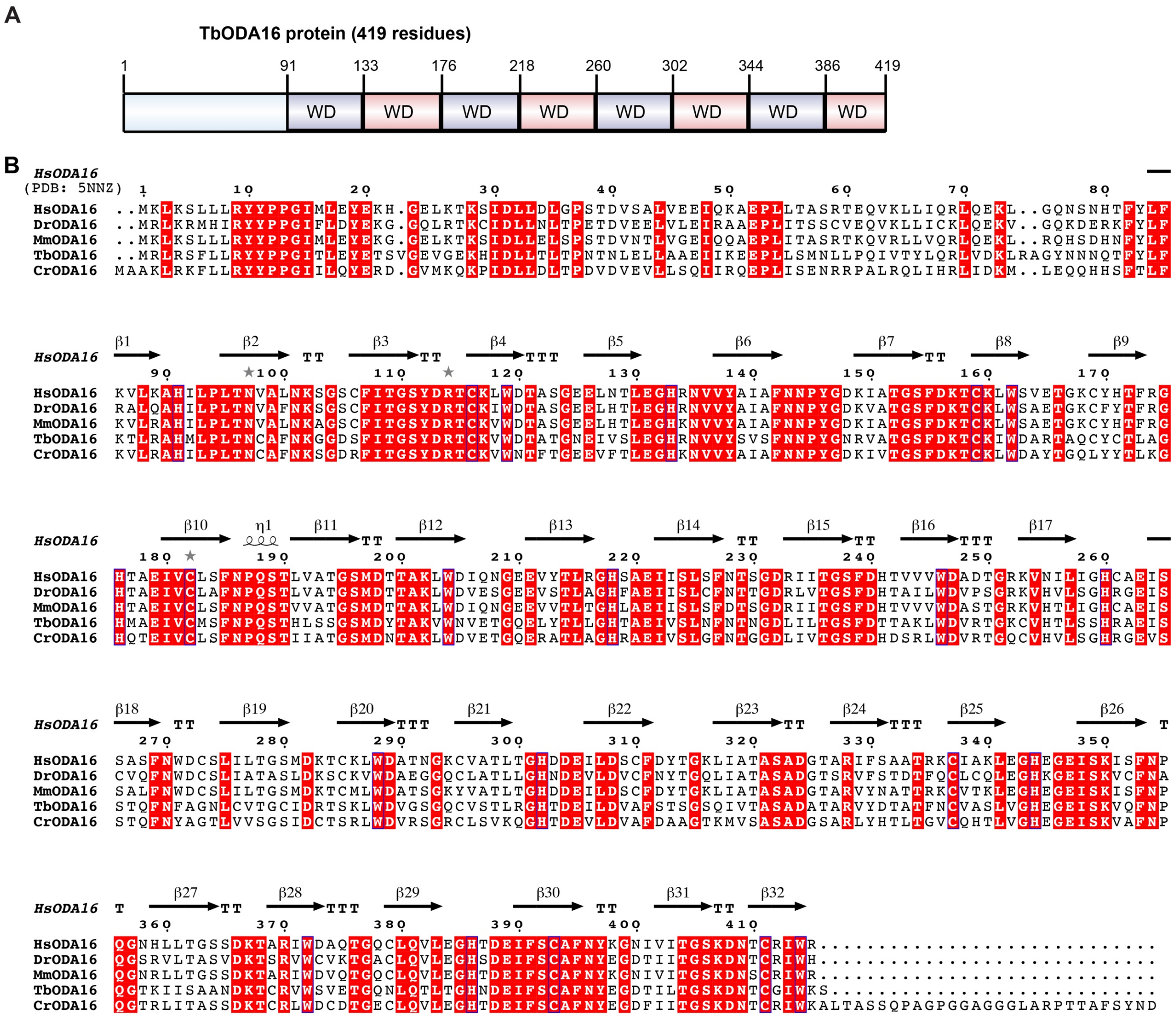

Fig. S2. Tb927.8.4210 encodes *T. brucei* homolog to ODA16.

(**A**) Tb927.8.4210 encodes a 419-aa polypeptide containing 8-bladed WD40 β-propeller domain. Domain prediction is retrieved from UniProt (Q57W14).

(**B**) Multiple sequence alignment of ODA16 homologues from indicated species. Secondary structures of HsODA16 (PDB: 5NNZ) are attached to the alignment as reference. Identical residues are boxed in red. Hs: *Homo sapiens*; Dr: *Danio rerio*; Mm: *Mus musculus*; Tb: *Trypanosoma brucei*; Cr: *Chlamydomonas reinhardtii*.

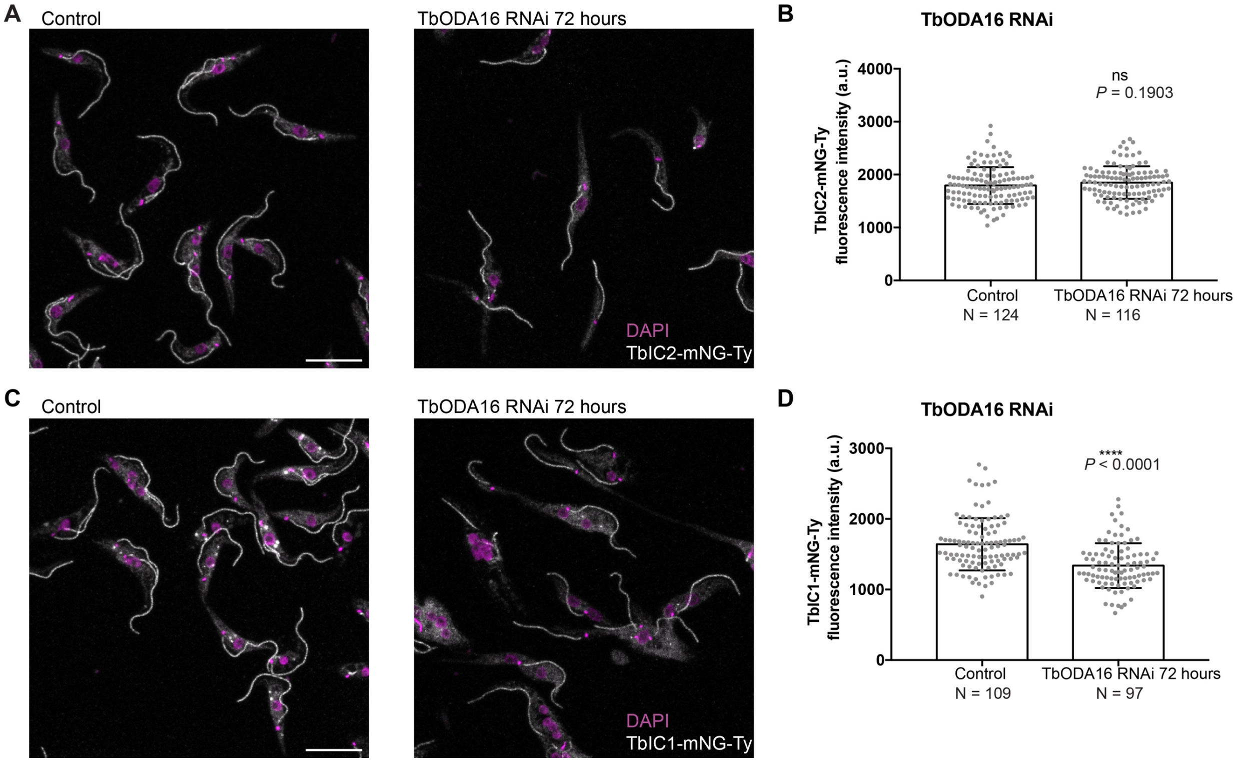

Fig. S3. The axonemal association of TbIC1 is partially reduced upon TbODA16 RNAi. (**A** and **C**) TbODA16 RNAi was induced in cells expressing mNG-Ty fusion to TbIC2 (A), TbIC1 (C). Scale bars: 10 µm. Their flagellar intensity was measured along the distal 1.5 µm and shown in (**B**) and (**D**), respectively. The results were shown as mean ± SD. *P* values were calculated by unpaired t test with Welch's correction.

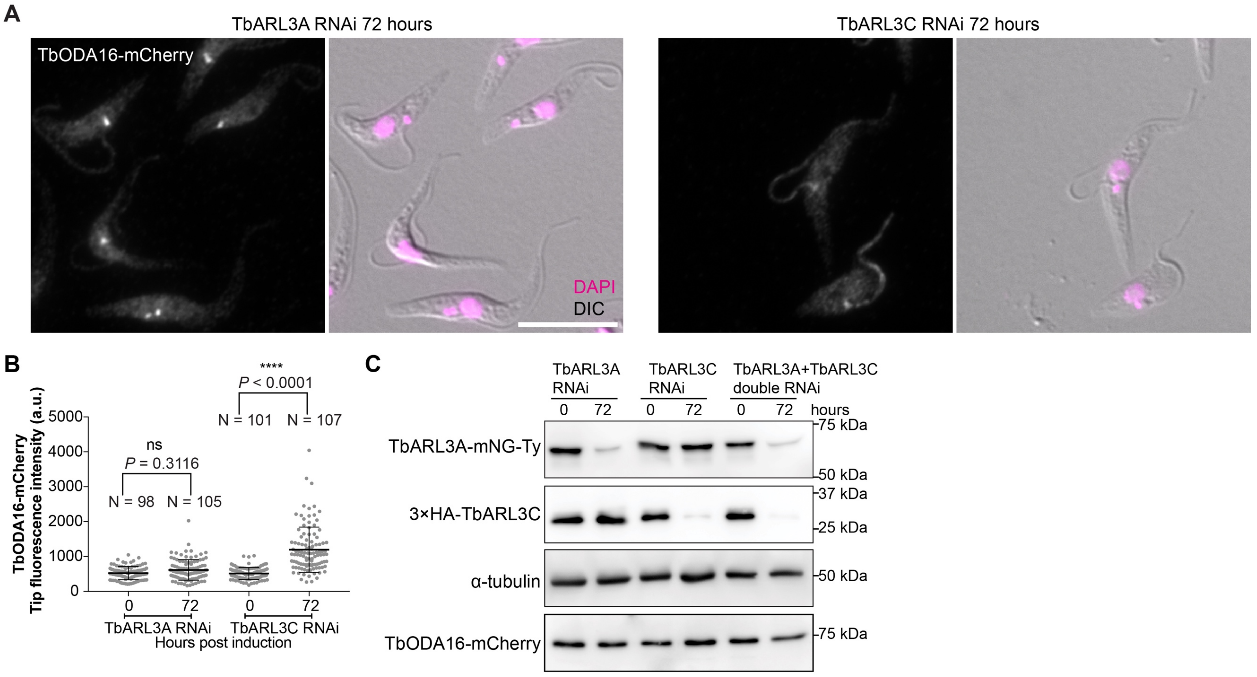

Fig. S4. Depletion of TbARL3C but not TbARL3A affects TbODA16 distribution.
(**A**) Immunofluorescence of cells with endogenously expressed TbODA16-mCherry, TbARL3A-mNG-Ty and 3×HA-TbARL3C upon induction for TbARL3A RNAi or TbARL3C RNAi for 72 hours. Scale bar: 10 µm.
(**B**) Comparison of TbODA16 intensity at the ciliary tip before and after RNAi of specified TbARL3 GTPases. The intensity was measured along the distal 1.5 µm of the flagellum. The results were shown as mean ± SD. *P* values were obtained from one-way ANOVA with Tukey's multiple comparisons test.
(**C**) Immunoblots confirming efficient and specific TbARL3 depletion in each of the RNAi cell lines shown in this figure as well as in Fig. 4A.

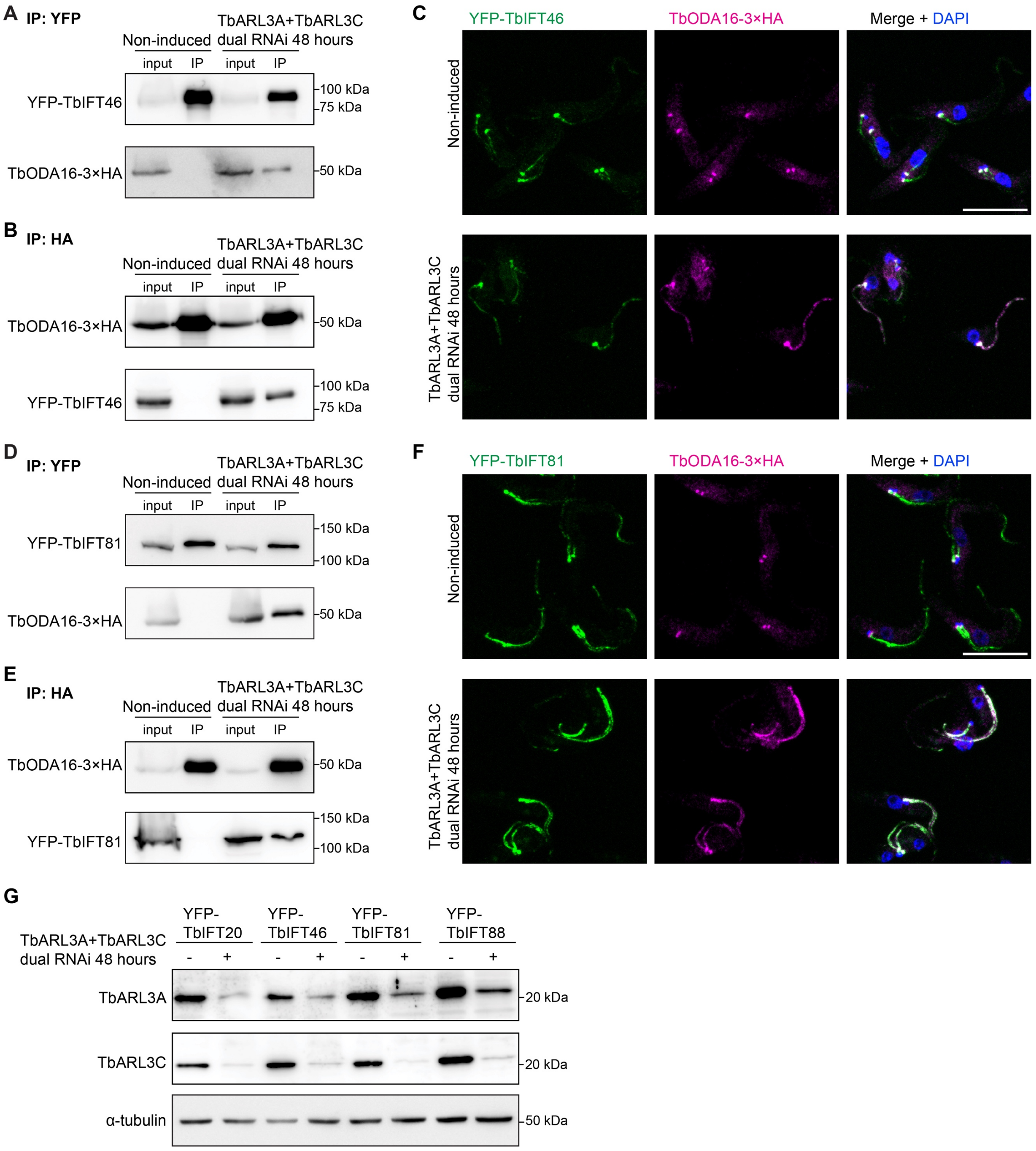

Fig. S5. Dual silencing of TbARL3A and TbARL3C stabilizes TbODA16-IFT interaction.

(**A-F**) Cells stably expressing HA-tagged TbODA16 and YFP-tagged IFT subunits IFT46 (A-C) or IFT81 (D-F) were induced for TbARL3A/TbARL3C dual RNAi or not. TbODA16-IFT interaction was assessed by co-IP using GFP-Trap (A and D) or anti-HA affinity gel (B and E). Immunofluorescence showing TbODA16 in control and TbARL3A/TbARL3C dual RNAi cells (C and F). Scale bars: 10 µm.

(**G**) Immunoblots confirming the depletion of TbARL3A and TbARL3C in all cell lines used in experiment shown in this figure and in Fig. 5.

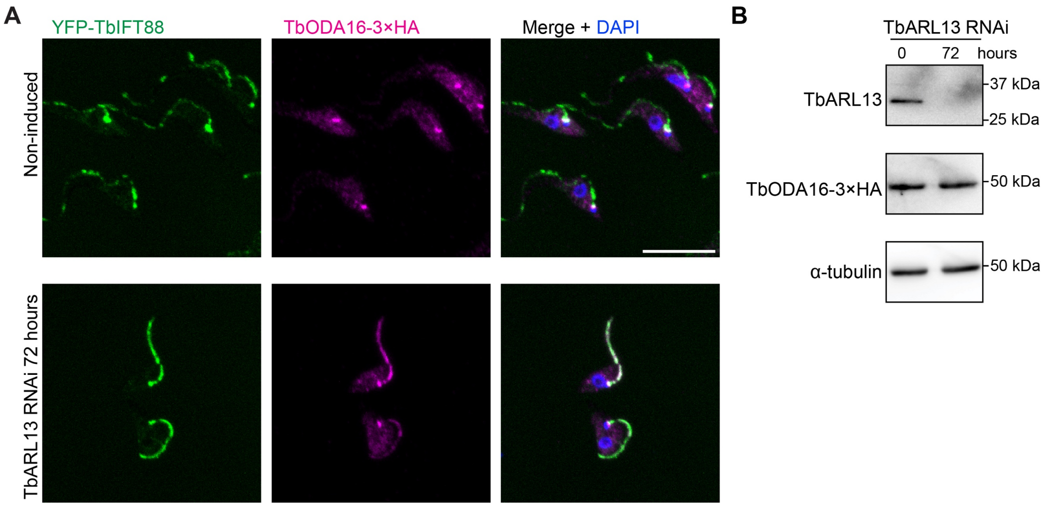

Fig. S6. Depletion of TbARL13 leads to TbODA16 accumulation in cilia.

(**A**) Representative immunofluorescence images of cells stably expressing HA-tagged TbODA16, induced for TbARL13 RNAi or not. Scale bar: 10 µm.

(**B**) Immunoblots showing depletion of TbARL13 protein, and unchanged expression of TbODA16 upon TbARL13 RNAi.

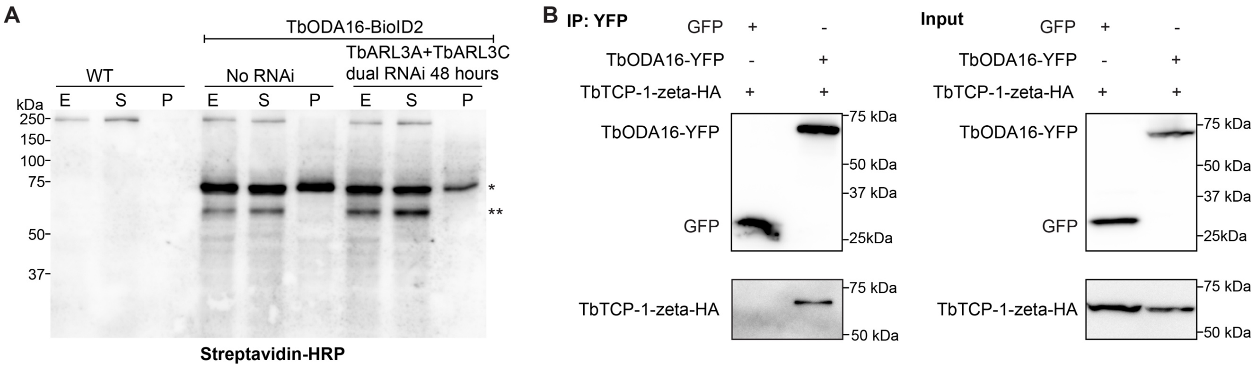

Fig. S7. TbODA16 is associated with TRiC.

(**A**) Immunoblots probed with streptavidin-HRP revealed the biotinylation profiles of TbODA16-BioID2. The cells were extracted with 1% NP40 in PEM buffer and centrifuged to obtain whole cell (E), detergent soluble (S) and detergent insoluble (P) fractions. WT, wild type cells not expressing TbODA16-BioID2 fusion; no RNAi, cells with expression of TbODA16-BioID2 but not induced for RNAi; TbARL3A+TbARL3C dual RNAi 48 hours, cells with expression of TbODA16-BioID2 and induced for TbARL3A/TbARL3C dual RNAi for 48 hours. * ~75kDa bands corresponding to the size of TbODA16-BioID2. ** ~60 kDa bands corresponding to the size of TRiC subunits.

(**B**) Co-IP assays confirming the interaction between TbODA16 and TRiC subunit TCP-1-zeta. Cells expressing GFP were used as a negative control.

Table S1. Synthetic DNA Sequences

*TbARL3A*iR	ATGGGACTTTTGACCTTGCTCAGAAAGCTTCGGAGCTCCGATGCC<br>AGCCCCAGAATCCTCATCTTGGGACTCGATAACGCAGGCAAGAC<br>CAGCATCCTGAGAAATCTGAGCGGAGAAGATCCCACAACCACAC<br>AGGCCACCCAAGGCTTTAATATCAAAACCGTGGACTGTGAGGGC<br>TTCAAGCTTAACGTCTGGGATATCGGCGGCCAGAAAGCAATAAG<br>AGCCTATTGGCCCAATTATTTCGATGAGGTCGATTGTCTTGTGTA<br>CGTCGTCGACAGCGCCGATAAAAGAAGACTCGATGAGACCGCTG<br>CAGAGCTCGAGACATTGTTACAGGAGGAGAAGTTGCGGGAGGTG<br>CCATTCTTGGTCTTGGCAAATAAATGTGACTTGGCCACCGCCTTA<br>AGCCCAGAAGATATCTCCACAGCTCTCAATTTGCAAAACCTCCG<br>GGACAGAACCTGGAGCATCCAGAAGTGTTCCGCTAAGACCGGCG<br>AAGGATTGCAAGAGGGATTTATGTGGGCTATCAAGTCCATCAAA<br>AAG
*TbARL3C*iR	ATGTTGAAAGGCATACGGTCCCGGGCCAAGCGGGACAACGAACC<br>CCGGGTCCTCATCGTCGGCTTGGATAATGCAGGCAAAACCACAG<br>TCCTCAACGCCCTTGGCGAGGATGAAGTCCCCGTCGAAGGCAAG<br>GTGAGCCATGCTGCCCCCGAAGGCCCCACCCAGGGCTTCAACAT<br>AAAGACCCTGACCCGGGGCAACAAGAGAGCAAAACTCTGCGAC<br>CTGGGCGGACAAAGAGCCCTCAGAGATTACTGGCAAGACTATTA<br>TTCCAATACCGATTGTATCATGTATGTCGTCGACAGCAGCGATCA<br>TAGAAGACTGGAGGAGAGCCATGCAGCCTTCGTCGACGTGTTAA<br>AAGGAATCGAGGGCGCACCCGTGCTTGTCTTCGCCAACAAGCAG<br>GACTTGGCCACAGCCAAGGACGCACAAGCCATAGCCGAGTGTTT<br>GCACCTTCACGATTTTAGAGATAGAAAATGGCATATACAGGGAT<br>GCTCCGCTAAGACCGGCGCCGGCCTCGAAGAGGGAGTCGCCTGG<br>ATCTTGTCCACCTGCGCTCCC
Synthetic *TbTCP-1-zeta* (aa376-544)	CGCAGTTGTACCCTTTTGGTGAAAGGTCCGAACGACCATACAAT<br>AGCGCAGCTGAAAGACGCGATACGAGACGGACTACGAGCTGTG<br>AAAAATGCATATGAACGCGGAGGTGTACTGGCTGGAGCAGGTTC<br>CTTCGAAGTCGCACTGCATGATCACCTCACGCGATACGCAGATA<br>CGGTCTCTGGCAAGCAGAAGATAGGAGTACGTGCCTATGCTGAC<br>GCGATACTCGTGATTCCCAAGACGCTCGCCGAAAATAGCGGGCT<br>AGACGTTCAACAATGTCTGATATCGCTCCAAGAAGCGAGTAGGC<br>GTGCTCGGCAAGAGGGCCGGTGGGTCGGGCTACGTCTGGATACC<br>GGTAGTACGGTAGATCCACTTGCTGCAGGCATTCTCGACAATGTG<br>CTAGTCAAGCGTAGCATCCTTGAAACCACAGGTGAAATAGTTGC<br>GCAACTTCTGCTAGTTGACGAGATCATGAAAGCAGGCCGTCGAG<br>GTGCTGGAGCGCCGCCATCCCAA

Movie S1. Live cell imaging and tracking of wild type *T. brucei* cells
Movie is presented at 6 frames per second. Scale bar: 10 μm.

Movie S2. Live cell imaging and tracking of *T. brucei* cells depleted of TbODA16
Movie is presented at 6 frames per second. Scale bar: 10 μm.

Movie S3. Live cell imaging and tracking of *T. brucei* cells depleted of TbARL3A and TbARL3C
Movie is presented at 6 frames per second. Scale bar: 10 μm.
